# A single-domain response regulator activates exopolysaccharide biosynthesis by interaction with the initiating phosphoglycosyl transferase

**DOI:** 10.1128/mbio.02986-25

**Published:** 2025-11-28

**Authors:** Johannes Schwabe, Julia Monjaras-Feria, Timo Glatter, Patrick Blumenkamp, Oliver Rupp, Alexander Goesmann, Miguel A. Valvano, Lotte Søgaard-Andersen

**Affiliations:** 1 Department of Ecophysiology, Max Planck Institute for Terrestrial Microbiology 28310, Marburg, Germany; 2 Infection Biology Group, Wellcome-Wolfson Institute for Experimental Medicine, Queen’s University Belfast, Belfast, United Kingdom; 3 Core Facility for Mass Spectrometry and Proteomics, Max Planck Institute for Terrestrial Microbiology 28310, Marburg, Germany; 4 Systems Biology and Bioinformatics, Justus Liebig University Gießen, Gießen, Germany; Indiana University Bloomington, Bloomington, Indiana, USA

**Keywords:** phosphoglycosyl transferase, polysaccharide biosynthesis, monoPGT, response regulator, proximity labeling, Wzx, Wzy

## Abstract

Exported polysaccharides play crucial functions in bacteria. Polysaccharide biosynthesis in the ubiquitous Wzx/Wzy- and ABC-transporter-dependent pathways starts with the transfer of a sugar-1-phosphate from a nucleotide-sugar donor to undecaprenyl phosphate, a reaction catalyzed by a phosphoglycosyl transferase (PGT). Both reaction substrates are limited and shared among multiple glycoconjugate pathways, raising the question of how bacteria regulate these pathways. In *Myxococcus xanthu*s, EpsZ, which belongs to the family of large monotopic PGTs (monoPGTs), starts the Wzx/Wzy-dependent exopolysaccharide (EPS) biosynthesis. The Dif chemosensory system regulates EPS biosynthesis by an unknown mechanism via the phosphorylated single-domain response regulator EpsW (EpsW~P). Here, we show that EpsW~P stimulates EPS biosynthesis at the post-translational level. Moreover, MiniTurbo-based proximity labeling experiments suggest that EpsW~P interacts directly with EpsZ. Additionally, heterologous expression of these two proteins in *Salmonella enterica* demonstrates that EpsW stimulates EpsZ enzymatic activity. *S. enterica* WbaP, the prototype large monoPGT, forms a functional homodimer, with dimerization involving a distinct cytoplasmic β-hairpin. However, AlphaFold-based structural modeling shows that EpsZ lacks this β-hairpin, suggesting an alternative mechanism for dimerization. Structural modeling of an EpsZ-EpsW heterocomplex suggests that EpsW~P, by direct interaction, promotes the formation of the stable EpsZ dimer. These findings suggest a new model for the regulation of polysaccharide biosynthesis in which EpsW~P allosterically facilitates the formation of the active, dimeric conformation of EpsZ, thereby activating EPS biosynthesis at its initial step. Genomics and structural modeling suggest that the regulation of large monoPGTs by a single-domain response regulator is widespread in myxobacteria and potentially beyond.

**IMPORTANCE:**

Bacteria produce various polysaccharides with important biological functions and biotechnological applications. Polysaccharide synthesis is energy-costly and requires substrates that are in limited supply, raising the question of how bacteria regulate these pathways. Here, we explored the regulation of exopolysaccharide biosynthesis in *Myxococcus xanthus*. We demonstrate that the phosphorylated single-domain response regulator EpsW activates exopolysaccharide biosynthesis at the post-translational level by stimulating the activity of the phosphoglycosyl transferase EpsZ. By interacting with EpsZ, phosphorylated EpsW facilitates the formation of the active, dimeric conformation of EpsZ, thereby activating exopolysaccharide biosynthesis at its initial step. We propose that this previously unrecognized regulatory mechanism is broadly conserved, not only in myxobacteria but also beyond.

## INTRODUCTION

Bacteria synthesize and export chemically diverse polysaccharides. These molecules contribute to biofilm formation, virulence, adhesion, motility, host-microbe interactions, and protection against phage infection and external stresses (1, 2) and also have applications in the food, biomedical, and pharmaceutical industries (3). Gram-negative bacteria synthesize and export these polysaccharides using three pathways: the Wzx/Wzy-, ABC transporter-, and synthase-dependent pathways (4–6). In the ubiquitous Wzx/Wzy- and ABC-transporter-dependent pathways, polysaccharide synthesis begins at the cytoplasmic leaflet of the inner membrane (IM) where an initiating phosphoglycosyl transferase (PGT) attaches a sugar-1-phosphate (sugar-1-P) from an activated nucleotide-sugar donor to undecaprenyl phosphate (Und-P) (7–9). The Und-PP-linked monosaccharide is the substrate for additional GTs that add monosaccharides and finalize the repeat unit in case of Wzx/Wzy-dependent pathways and the entire polysaccharide in case of ABC-transporter-dependent pathways (4). The monotopic PGTs (monoPGTs) constitute a prevalent PGT superfamily that shares a conserved catalytic domain (10, 11). MonoPGTs are further divided into small and large monoPGTs (9). Small monoPGTs, of which PglC from *Campylobacter concisus* (PglC*_Cc_*) is the prototype (11, 12), are monomeric and consist of only the catalytic domain (9, 13). By contrast, the large monoPGTs are dimeric and possess three conserved domains: a bundle of four transmembrane α-helices (TMHs), a cytoplasmic domain of unknown function (DUF), and the C-terminal catalytic domain (9, 14, 15). The *Salmonella enterica* WbaP (WbaP*_Se_*), which transfers galactose-1-phosphate (Gal-1-P) to UndP, is the prototype of this family (16–20).

In the Wzx/Wzy-dependent pathways, the Wzx flippase translocates the Und-PP-linked repeat units across the IM into the periplasm, where the Wzy polymerase catalyzes their polymerization (4) by a process involving the polysaccharide co-polymerase (PCP) (4, 21). In pathways for capsular and secreted polysaccharides, the Und-PP-linked polysaccharides are exported across the outer membrane (OM) via a cell-envelope spanning export complex comprising either the PCP and an OM polysaccharide export (OPX) protein (4, 21) or a PCP, a periplasmic OPX protein, and an integral 16- to 18-stranded OM β-barrel protein (22, 23). Alternatively, in LPS biosynthetic pathways, the Und-PP-linked O-antigen polysaccharide is transferred to the lipid A-core oligosaccharide by the WaaL ligase (24) and transported to the cell surface by the Lpt lipopolysaccharide export pathway (25).

Polysaccharide biosynthesis via Wzx/Wzy-dependent pathways is energy-costly and also draws UndP from a limited supply that must be sustained for the biosynthesis of other glycoconjugates, including the essential peptidoglycan (26–31). Accordingly, these pathways are regulated, commonly at the transcriptional level (32–38), but post-translational regulation has also been described, i.e., in *Staphylococcus aureus,* the bacterial tyrosine kinase CapB1 phosphorylates the small monoPGT CapM, increasing its catalytic activity *in vitro* (39), and phosphoproteome analysis has revealed that the *Klebsiella pneumoniae* large monoPGT WcaJ is Tyr-phosphorylated, albeit a functional analysis of this modification is lacking (40). While the post-translational mechanisms of regulation of Wzx/Wzy-dependent pathways are incompletely understood, the post-translational regulation of synthase-dependent pathways is well characterized, frequently involving the second messenger c-di-GMP (6). In these cases, c-di-GMP can stimulate synthase activity by binding to an allosteric site (41), to a non-catalytic partner protein of the synthase (42–44), or by enabling the interaction of the catalytic domain and a non-catalytic partner protein (45).

**Fig 1:**
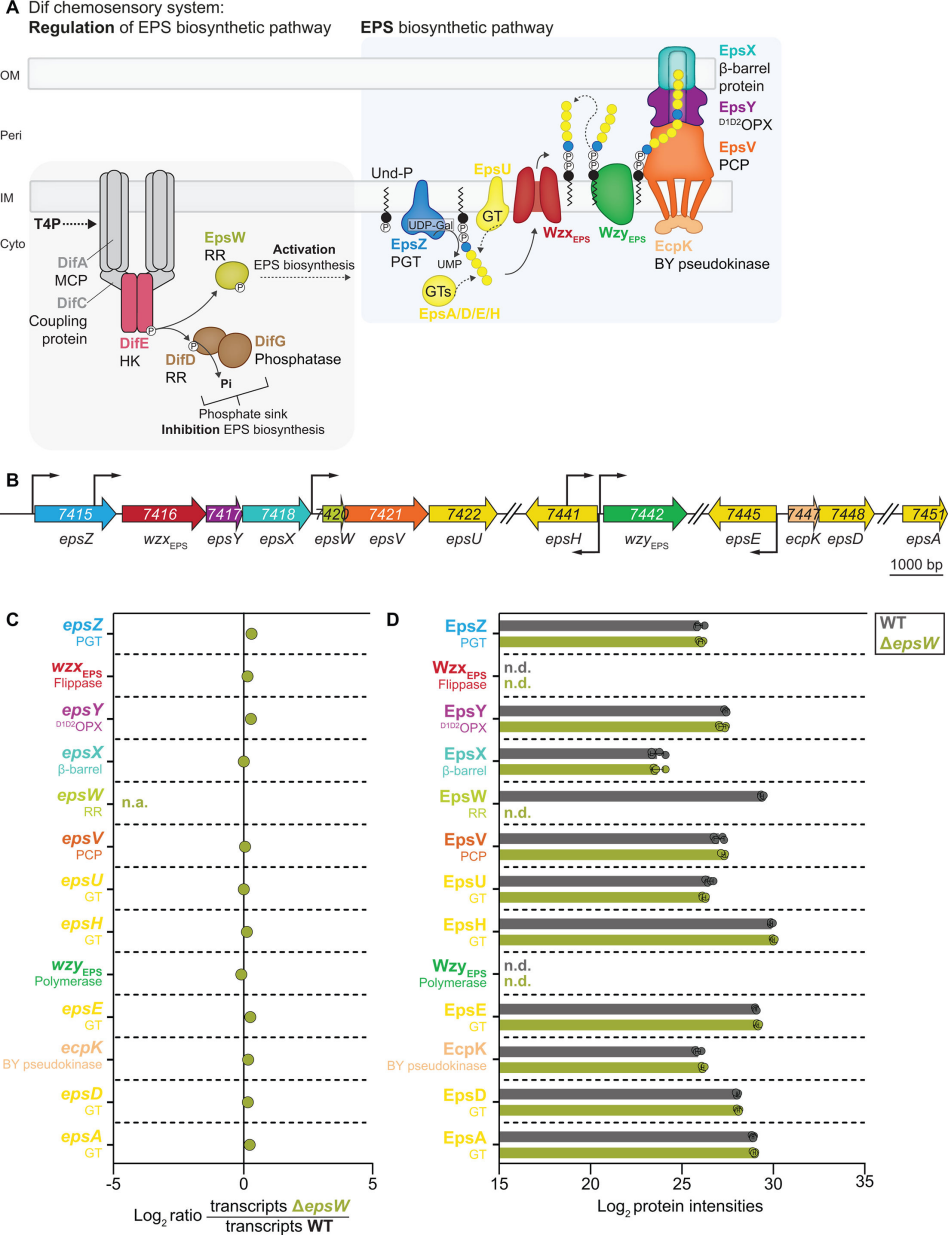
The Eps proteins accumulate independently of EpsW. (**A**) Schematic of the Dif chemosensory system and the EPS biosynthetic pathway in *M. xanthus*. Stippled line, mechanism unknown; continuous line, mechanism known. See text for details. MCP, methyl-accepting chemotaxis protein; HK, histidine kinase; RR, response regulator. EpsZ transfers Gal-1-P to Und-P. The repeat units are built by several GTs and translocated into the periplasm by the Wzx_EPS_ flippase. The Wzy_EPS_ polymerase builds the final EPS molecule. EpsV is the PCP and functions together with the bacterial tyrosine pseudokinase (BY pseudokinase) EcpK (49). EPS is exported across the OM through a bipartite translocon comprising the ^D1D2^OPX protein EpsY, an OM polysaccharide export protein only comprising the periplasmic domains D1 and D2, and the 18-stranded OM β-barrel protein EpsX (22, 23). (**B**) Operon structure of the two gene clusters for *eps* biosynthesis. Kinked arrows, transcription start sites (60). Numbers, MXAN_locus tags. EpsW, while a downstream target of the Dif chemosensory system, is encoded in the *eps* locus. (**C**) Differential expression of *eps* genes in the Δ*epsW* mutant compared to wild type (WT). The RNA sequencing experiment was performed with four biological replicates per strain from cells grown in suspension culture. *X*-axis, log_2_-fold ratio of the mean transcripts in the Δ*epsW* mutant over the mean transcripts in the WT calculated using the DESeq2 method (61). n.a., non-applicable. Statistical analysis was performed in the DeSeq2 analysis. No significant differences were identified (adjusted *P-*value ≤ 0.001). (**D**) Protein abundance in whole-cell proteomes of the Δ*epsW* mutant compared to WT. The label-free quantitative mass spectrometry-based proteomics was performed with four biological replicates per strain from cells grown as in panel **C**. *X*-axis, normalized log_2_ intensities of Eps proteins. Data points represent each of the four biological replicates. Error bars, standard deviation. No significant differences were identified (Welch’s test, *P-*value ≤ 0.001). n.d., not detected.

In the Gram-negative bacterium *Myxococcus xanthus*, the exopolysaccharide (EPS), which is synthesized and exported by the Wzx/Wzy-dependent EPS pathway (22, 23, 46–49), is crucial for type IV pili (T4P)-dependent motility, adhesion, development, and biofilm formation (50). EPS synthesis starts with the transfer of Gal-1-P to Und-P, catalyzed by the large monoPGT EpsZ (46) (Fig. 1A). EPS biosynthesis is regulated by the Dif chemosensory system (51–53) via the phosphorylated single-domain response regulator EpsW (54) (Fig. 1A). The DifE histidine protein kinase phosphorylates EpsW (54), but how phosphorylated EpsW (EpsW~P) stimulates EPS biosynthesis is unknown. DifE also phosphorylates the single-domain response regulator DifD, which, together with the DifG phosphatase, acts as a phosphate sink competing with EpsW for phosphorylation by DifE to inhibit EPS biosynthesis (55, 56) (Fig. 1A). The signals regulating Dif activity are unknown, but it has been suggested that T4P extension activates the Dif system (57–59).

Here, we focused on how EpsW~P activates EPS biosynthesis. Global transcriptomic and proteomic analyses indicated that EpsW regulates EPS biosynthesis at the post-translational level. MiniTurbo-based proximity labeling experiments in *M. xanthus* strongly suggested that EpsW~P directly interacts with EpsZ. Additionally, *in vivo* heterologous expression experiments in *S. enterica* demonstrated that while EpsZ alone had low Gal-1-P transferase activity, this activity was strongly stimulated when co-expressed with EpsW, suggesting that EpsW~P, by direct interaction, brings about the active EpsZ conformation. Computational structural analyses using AlphaFold indicated that while EpsZ alone may be compromised in dimer formation, EpsW, by directly interacting with EpsZ, promotes the formation of the stable EpsZ dimer. Together, these findings support a previously undescribed allosteric mechanism whereby a single-domain response regulator, by direct interaction, stimulates the enzymatic activity of a large monoPGT by facilitating its active, dimeric conformation.

## RESULTS

### EpsW activates EPS biosynthesis at the post-translational level

Single-domain response regulators can function in phosphorelays to regulate gene expression or engage in protein-protein interactions to control the activity of a downstream protein (complex) (62, 63). To explore how EpsW stimulates EPS biosynthesis, we identified differentially expressed genes and differentially accumulating proteins by RNA sequencing (RNA-seq) and whole-cell, label-free quantitative mass spectrometry-based proteomics in the Δ*epsW* mutant and the parental wild-type (WT) strain. In these experiments, cells were grown exponentially in suspension, a condition where *M. xanthus* synthesizes and exports EPS (46, 64).

We first analyzed genes and proteins known to be involved in EPS biosynthesis or its regulation, including the core genes/proteins for T4P formation and function. RNA transcript levels of all *eps* genes (Fig. 1B and C), *dif* genes (Fig. S1A), other genes encoding regulators of EPS biosynthesis (Fig. S1B), and genes for the core proteins for T4P formation and function (Fig. S2A) were similar in WT and the Δ*epsW* mutant. The corresponding proteins detected by whole-cell proteomics accumulated at similar or slightly higher levels in the Δ*epsW* mutant compared to WT (Fig. 1D; Fig. S1C, D, and S2B). These findings suggest that EpsW does not activate EPS biosynthesis by regulating the abundance of Eps and Dif proteins, other regulators of EPS biosynthesis, or the core proteins for T4P formation and function.

T4P extension requires a priming complex made of four minor pilins and the PilY1 adhesin (65). The *M. xanthus* genome contains three gene clusters encoding distinct sets of minor pilins/PilY1 proteins (65). Cluster_1 alone and cluster_3 alone are sufficient for T4P formation, while cluster_2 does not contribute to T4P formation, and the corresponding proteins do not detectably accumulate (65). RNA transcripts of all *cluster_1*, *cluster_2,* and *cluster_3* genes were detected in the WT (Fig. S3A), with low levels of *cluster_2*, as exemplified by *pilY1.2* (Fig. S3B). In the Δ*epsW* mutant, the transcript levels of *cluster_1* genes were as in WT, except for *pilX1,* which was slightly but significantly reduced (Fig. S3A). The transcript levels of all five *cluster_3* genes and two *cluster_2* genes were also significantly reduced in the Δ*epsW* mutant (Fig. S3A). The transcript levels of *hsfA* and *hsfB*, encoding a phosphorelay that regulates transcription of *cluster_1* and *cluster_3* expression (66), were slightly but significantly increased in the Δ*epsW* mutant (Fig. S3A). However, whole-cell proteomics revealed significantly reduced abundance of only two cluster_3 proteins, while the abundance of HsfA was slightly increased (Fig. S3C). To assess whether these changes in the abundance of cluster_3 minor pilins could cause the EPS biosynthetic defect in the Δ*epsW* mutant, we determined EPS biosynthesis in different cluster mutants (Fig. S3D). While the Δ*cluster_2*Δ*cluster_3* double mutant synthesized EPS at WT levels, the Δ*cluster_1*Δ*cluster_2*Δ*cluster_3* triple mutant, which lacks all genes encoding the three priming complexes, did not synthesize EPS (Fig. S3D). Thus, cluster_1 is sufficient for WT levels of EPS biosynthesis, arguing that the EPS biosynthesis defect in the Δ*epsW* mutant is not due to the reduced accumulation of two of the cluster_3 proteins.

We also investigated changes at the global transcript and protein levels in the Δ*epsW* mutant compared to the WT (Materials and Methods). Except for the *cluster_3* genes (Fig. S3A), only two genes encoding hypothetical proteins had reduced transcript levels (Table S1). Four genes, encoding CRISPR-associated proteins, had increased transcript levels (Table S1). A total of 22 and 5 proteins had reduced and increased accumulation levels, respectively (Table S2). However, none of these genes and proteins have been implicated in EPS biosynthesis.

Collectively, these findings suggest that the EPS biosynthetic defect in the absence of EpsW does not depend on altered gene expression or differences in protein abundance, indicating that EpsW stimulates EPS biosynthesis at the post-translational level.

### Phosphorylation of EpsW enhances the EpsW-EpsZ interaction *in vivo*

We speculated that EpsW could stimulate EPS biosynthesis post-translationally by engaging in direct protein-protein interactions. Therefore, we searched for EpsW interaction partners *in vivo* by proximity labeling using EpsW fused to the biotin ligase miniTurbo (mTurbo) (67–70). We ensured that mTurbo-based proximity labeling could detect Eps proteins and DifE (the only known interaction partner of EpsW) by assessing the presence of surface-accessible cytoplasmic Lys residues in these proteins. Manual counting using AlphaFold2-generated models revealed that all Eps proteins and DifE, except for EpsY and EpsX, which lack cytoplasmic domains (Fig. 1A) (22, 23), have Lys residues amenable to biotinylation (Table S3).

For the biotinylation experiments, we ectopically expressed *mTurbo-epsW-FLAG* from the *pilA* promoter in three different mutants: Δ*epsW*, Δ*difE*Δ*epsW* (lacking the DifE kinase that phosphorylates EpsW), and Δ*difD*Δ*difG*Δ*epsW* (lacking DifD and the DifG phosphatase that jointly function as a phosphate sink for the phosphoryl groups of DifE) (Fig. 1A). As controls for unspecific biotinylation, we co-expressed sfGFP-mTurbo-FLAG from the vanillate-inducible promoter (67) and untagged EpsW from the *pilA* promoter in the three mutants. mTurbo-EpsW-FLAG and sfGFP-mTurbo-FLAG (in the presence of 7.5 µM vanillate) accumulated at similar levels, and both had biotin-ligase activity in all three strains (Fig. S4A through D). Moreover, mTurbo-EpsW-FLAG restored EPS biosynthesis and T4P-dependent motility in the Δ*epsW* and the Δ*difD*Δ*difG*Δ*epsW* mutants, with the Δ*difD*Δ*difG*Δ*epsW* strain producing slightly more EPS (Fig. S5), demonstrating that the fusion protein is functional and there is enhanced phosphate flux from DifE to mTurbo-EpsW-FLAG in the absence of DifD/DifG. As expected, mTurbo-EpsW-FLAG supported neither EPS biosynthesis nor T4P-dependent motility in the Δ*difE*Δ*epsW* strain (Fig. S5). Similarly, the control strains co-expressing untagged EpsW and sfGFP-mTurbo-FLAG in the Δ*epsW* and the Δ*difD*Δ*difG*Δ*epsW* strains had EPS biosynthesis and T4P-dependent motility, again with slightly more EPS biosynthesis in the Δ*difD*Δ*difG*Δ*epsW* strain (Fig. S5). As expected, the Δ*difE*Δ*epsW* strain co-expressing untagged EpsW and sfGFP-mTurbo-FLAG did not have EPS biosynthesis and T4P-dependent motility (Fig. S5).

**Fig 2:**
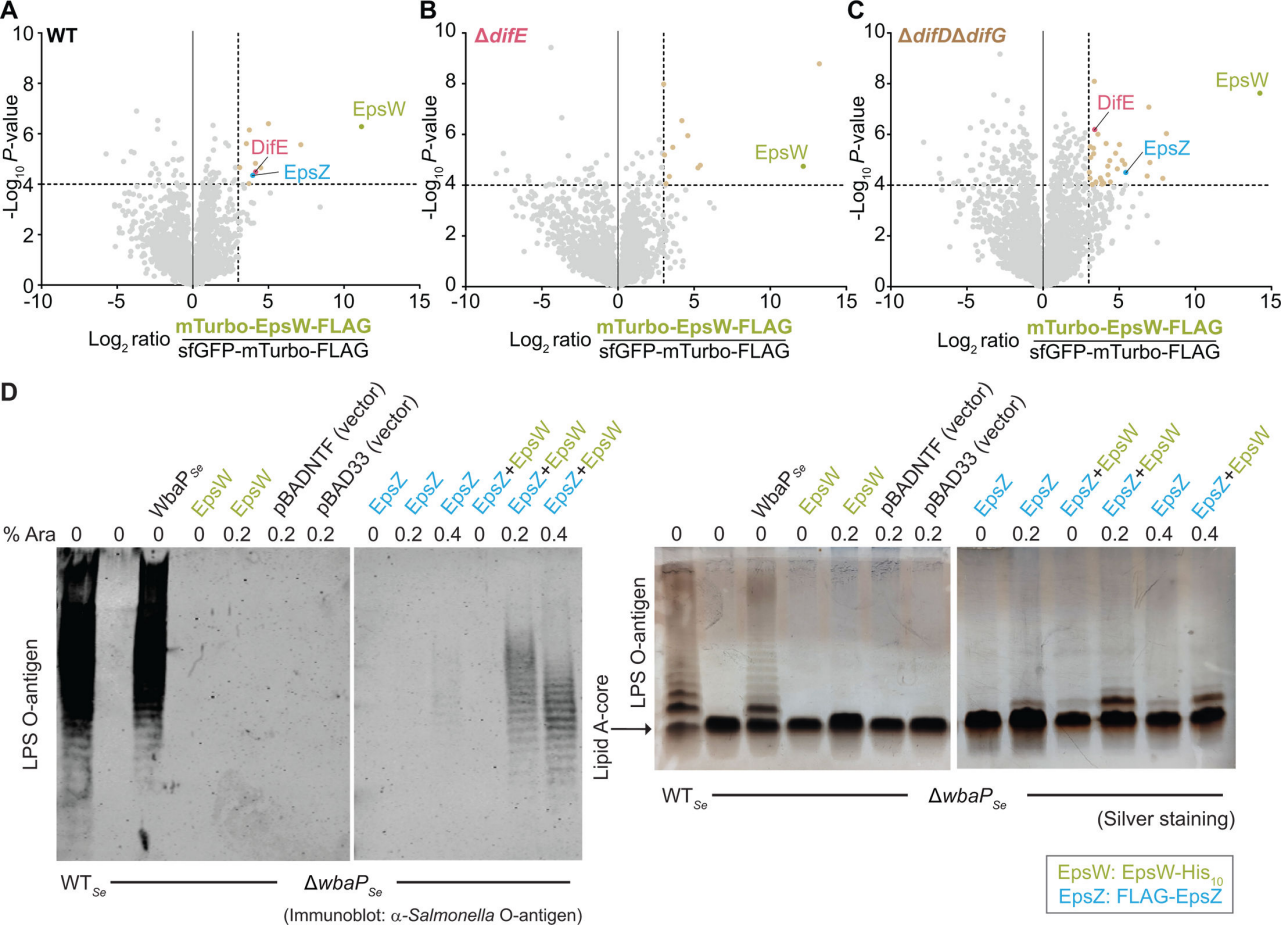
EpsW phosphorylation enhances the EpsW-EpsZ interaction *in vivo* and EpsW stimulates EpsZ Gal-1-P transferase activity. (**A–C**) Volcano plots showing the enrichment of proteins in the proximity labeling experiment with mTurbo-EpsW-FLAG and sfGFP-TurboID-FLAG variants in the otherwise WT (**A**), Δ*difE* (**B**)*,* and Δ*difD*Δ*difG* (**C**) strains. *mTurbo-epsW-FLAG* was expressed from the *pilA* promoter on a plasmid integrated in a single copy at the Mx8 *attB* site. In the *sfGFP-mTurbo-FLAG* strains, *epsW* was expressed from the *pilA* promoter on a plasmid integrated in a single copy at the Mx8 *attB* site, and *sfGFP-mTurbo-FLAG* was expressed under the control of the vanillate-inducible promoter from a plasmid integrated in a single copy at the *18-19* intergenic locus. For all strains, samples were prepared from four biological replicates. *X*-axis, log_2_-fold ratios of mean protein intensities in mTurbo-EpsW-FLAG samples relative to the sfGFP-mTurbo-FLAG samples. *Y*-axis, −log_10_ *P*-value. Significantly enriched proteins fulfill the criteria log_2_ fold ratio ≥ 3.0 and −log_10_ *P-*value ≥ 4.0 and are indicated by wheat-colored data points and listed in Tables S4 to S6. Significance thresholds are indicated with dashed lines. Data points represent the means of four biological replicates. (**D**) EpsW stimulates EpsZ Gal-1-P transferase activity. Complementation of the *S. enterica* Δ*wbaP* mutant (Δ*wbaP_Se_*) by ectopic expression of the indicated proteins. LPS samples were extracted from the indicated strains and examined by immunoblotting using α-*Salmonella* O-antigen antiserum (left) and silver staining (right). Samples from an equal number of cells were loaded per sample. In both analyses, samples were prepared, separated on two gels, and probed with antibodies/silver-stained in parallel. Arabinose (Ara) was added as indicated. FLAG-EpsZ and EpsW-His_10_ were expressed from pMP146 (vector: pBADNTF) and pJSc143 (vector pBAD33), respectively, under the control of an arabinose-inducible promoter. WbaP*_Se_* was expressed from pSM13 under the control of a constitutive *lac* promoter.

In the proximity labeling experiments in the WT background (Fig. 2A; Table S4), 11 proteins, including EpsW, were significantly enriched in the mTurbo-EpsW-FLAG samples, indicating successful biotinylation and enrichment of the fusion protein. Importantly, the DifE kinase, the only known EpsW interaction partner, was enriched. Strikingly, EpsZ was also enriched. None of the remaining eight proteins (Table S4), including the glycosyl hydrolase (family 57) MXAN_2994 and the GNAT-family acetyltransferase MXAN_4576 that may participate in monosaccharide synthesis or modification, have been identified as important for EPS biosynthesis.

In the Δ*difE* mutant (no EpsW phosphorylation), 11 proteins, including EpsW, but not EpsZ, were significantly enriched (Fig. 2B; Table S5). None of the remaining 10 proteins have been identified as important for EPS biosynthesis (Table S5).

In the absence of DifD and DifG (increased EpsW phosphorylation), 33 proteins, including EpsW, DifE, and EpsZ, were significantly enriched (Fig. 2C; Table S6), with EpsZ enrichment even more prominent (log_2_-fold increase of 1.4 compared to EpsZ enrichment in the WT strain). None of the remaining proteins, including the GNAT-family acetyltransferase MXAN_2367, have been identified as important for EPS biosynthesis (Table S6).

Among the four proteins that were enriched in both WT and Δ*difD*Δ*difG* strains but not in the Δ*difE* strain (Tables S4 to S6), only DifE and EpsZ were reported to function in EPS biosynthesis, and the two remaining proteins are not predicted to function in polysaccharide biosynthesis. Because the labeling radius of mTurbo *in vivo* is ∼10 nm (71), these results indicate that upon phosphorylation by DifE, EpsW is in close proximity to and interacts with EpsZ. Because an EpsW variant with a substitution of the phosphorylatable Asp residue (D^58^) does not stimulate EPS biosynthesis (54), these observations imply that EpsW~P stimulates EPS biosynthesis by interacting with EpsZ.

### EpsW activates EpsZ Gal-1-P transferase activity in a heterologous system

We used an *S. enterica*-based heterologous expression system to test whether EpsW stimulates EpsZ Gal-1-P transferase activity *in vivo*. In *S. enterica*, WbaP*_Se_* initiates the synthesis of the LPS O-antigen by catalyzing the transfer of Gal-1-P to Und-P (19). Using LPS O-antigen biosynthesis as a readout, we previously showed that EpsZ with an N-terminal FLAG-tag (FLAG-EpsZ) weakly complements the *S. enterica* Δ*wbaP* (Δ*wbaP_Se_*) mutant, but these earlier experiments did not include EpsW (46).

Here, we expressed FLAG-EpsZ without or with an active EpsW-His_10_ variant (Fig. S6A and B) in the Δ*wbaP_Se_* mutant. As a positive control, we initially confirmed that the ectopic expression of WbaP restored LPS O-antigen synthesis in this mutant (Fig. 2D). As previously observed, FLAG-EpsZ alone, expressed from an arabinose-inducible promoter, only weakly complemented the LPS O-antigen biosynthetic defect in the presence of 0.4% arabinose, as shown in immunoblots with *Salmonella* O-antigen-specific antibodies and by silver staining (46) (Fig. 2D). As expected, EpsW-His_10_, which was also expressed from an arabinose-inducible promoter, did not restore LPS O-antigen synthesis (Fig. 2D). By contrast, co-expression of FLAG-EpsZ and EpsW-His_10_ in the presence of 0.2% and 0.4% arabinose efficiently restored LPS O-antigen biosynthesis in the Δ*wbaP_Se_* mutant (Fig. 2D). Immunoblots using α-FLAG antibodies demonstrated that FLAG-EpsZ abundance correlated with the arabinose concentration and was independent of the presence or absence of EpsW-His_10_ (Fig. S6C). Under the conditions used for protein denaturation and SDS-PAGE, FLAG-EpsZ accumulated predominantly in the monomeric form and less as higher molecular weight oligomers in both strains, while other large monoPGTs display a more equal distribution between the monomer and the oligomers (20, 72, 73). In immunoblots, we were unable to detect EpsW-His_10_ in the Δ*wbaP_Se_* mutant using α-His_6_ antibodies.

We conclude from these results that EpsW stimulates EpsZ Gal-1-P transferase activity *in vivo*. In *M. xanthus*, EpsW-dependent stimulation of EPS biosynthesis depends on the DifE-dependent phosphorylation (54). Therefore, we speculate that EpsW in the Δ*wbaP_Se_* mutant is either non-specifically phosphorylated by a *S. enterica* histidine protein kinase or autophosphorylates using low molecular weight phosphodonors like acetyl phosphate or carbamoyl phosphate (74, 75). Because EpsW~P is in close proximity to and likely interacts with EpsZ in *M. xanthus*, these observations suggest that EpsW~P enhances EpsZ Gal-1-P transferase activity by direct interaction.

### EpsZ has a similar catalytic domain as prototypic PGTs but lacks a characteristic β-hairpin in the cytoplasmic DUF

Our results suggest that EpsW~P is a direct activator of EpsZ enzymatic activity. However, the activity of the PglC*_Cc_* and WbaP*_Se_* prototype PGTs does not depend on regulatory proteins, raising the question of the EpsZ-specific mechanism of activation. To understand the mechanism of EpsW~P-mediated EpsZ activation, we performed structural modeling in three steps.

**Fig 3:**
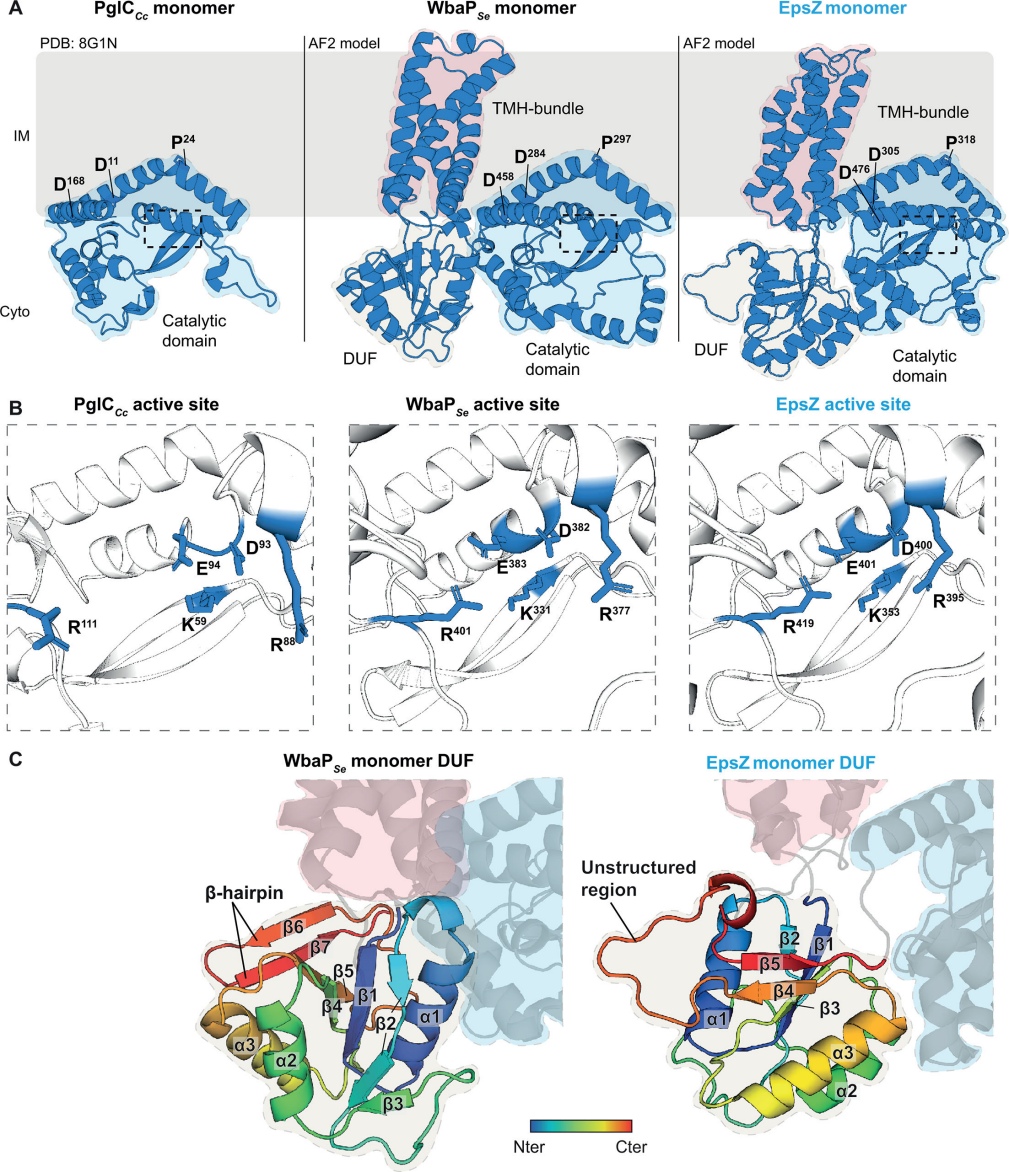
EpsZ is a large monoPGT lacking a characteristic β-hairpin in the cytoplasmic DUF. (**A**) Computational structural comparison of EpsZ with the prototype monoPGTs PglC*_Cc_* and WbaP*_Se_*. Left panel, solved structure of the small monoPGT PglC*_Cc_* (11). Middle and right panels, rank 1 AlphaFold2 models of monomeric WbaP*_Se_* and monomeric EpsZ. All panels, regions in light blue indicate the catalytic domain. Regions in silk and light red in the middle and right panels indicate the cytoplasmic DUF and the TMH bundle. Conserved residues important for the re-entrant α-helix architecture are highlighted; see text for details. The stippled boxes mark the position of the active site. Protein orientation in the IM is based on computational analysis using the PPM server (76). (**B**) Computational structural comparison of the active sites of EpsZ with those of PglC*_Cc_* and WbaP*_Se_.* Left panel, active site of the solved structure of PglC*_Cc_* (11). Middle and right panels, active sites of the rank 1 AlphaFold2 models of monomeric WbaP*_Se_* and monomeric EpsZ. Conserved residues important for catalytic activity are highlighted (11, 13, 16). (**C**) Computational structural comparison of the cytoplasmic DUF of EpsZ with that of WbaP*_Se_*. Left and right panels, cytoplasmic DUF of the rank 1 AlphaFold2 models of monomeric WbaP*_Se_* and EpsZ. In both panels, the DUF is shown using a gradient from blue (N-terminus) to red (C-terminus). Numbering of α-helices and β-sheets is included. In both panels, regions colored in blue and red indicate the catalytic domain and the TMH bundle.

First, we generated AlphaFold2 models of monomeric WbaP*_Se_* and EpsZ. The WbaP*_Se_* model had high confidence (Fig. S7A and B), revealing a structure consisting of the catalytic domain, TMH bundle, and cytoplasmic DUF (Fig. 3A), as previously described (14, 20). The EpsZ model, also of high confidence (Fig. S7A and C), contained equivalent domains (Fig. 3A).

In monoPGTs, the conserved catalytic domain integrates into the IM via the re-entrant α-helix, which is kinked by a Pro residue and enters and exits the IM’s lipid bilayer at the cytoplasmic leaflet (10, 11). This Pro residue is part of the D_X12_P motif, which is strictly conserved in monoPGTs (77), and is also present in PglC*_Cc_*, WbaP*_Se_,* and EpsZ (10, 11, 46). In the WbaP*_Se_* and EpsZ structural models, the D_X12_P motif mapped to similar positions (D^284^ and P^297^ in WbaP*_Se_* and D^305^ and P^318^ in EpsZ) on the re-entrant α-helix as in the catalytic domain of PglC*_Cc_* (D^11^ and P^24^) (Fig. 3A). Additionally, a strictly conserved Asp residue suggested to function in stabilizing the re-entrant αhelix in PglC*_Cc_* (D^168^) (11, 77) mapped to equivalent positions in WbaP*_Se_* (D^458^) and EpsZ (D^476^) (Fig. 3A).

In PglC*_Cc_*, the active site involves a catalytic dyad (D^93^E^94^) and additional residues for substrate orientation (K^59^, R^88^, and R^111^) (11) that are conserved among monoPGTs (77) (Fig. 3B). The WbaP*_Se_* and EpsZ structural models had active site architectures comparable to that of PglC*_Cc_* (Fig. 3B). This agrees with the current model that the catalytic mechanism of monoPGTs is conserved irrespective of the nucleotide-sugar substrates (77–79). Based on these comparisons, we conclude that the activation of EpsZ is not related to differences in its catalytic domain.

Because the previous results suggested that the EpsZ-specific mechanism for activation does not involve the catalytic domain, we compared the cytoplasmic DUF of EpsZ and WbaP*_Se_*. The WbaP*_Se_* DUF model has a central β-sheet composed of five β-strands (β1–β5), with β2 interrupted by a short unstructured region (Fig. 3C). Three α-helices surround the central β-sheet (Fig. 3C). Additionally, the C-terminal region contains a β-hairpin built by the β6/β7-strands (Fig. 3C). Despite the cytoplasmic DUF in EpsZ having a superficially similar fold to its WbaP*_Se_* counterpart, with both featuring a central β-sheet built of five β-strands surrounded by three α-helices, (Fig. 3C), three key differences exist: (i) unlike WbaP*_Se_*, the EpsZ cytoplasmic DUF lacks the C-terminal β-hairpin (Fig. 3C), (ii) the β4/β5-strands are separated by an unstructured region (Fig. 3C), and (iii) the α2/α3-helices are oriented toward the catalytic domain, whereas in WbaP*_Se_*, it is the α1-helix that faces the catalytic domain.

### EpsZ alone might be compromised in dimer formation

**Fig 4:**
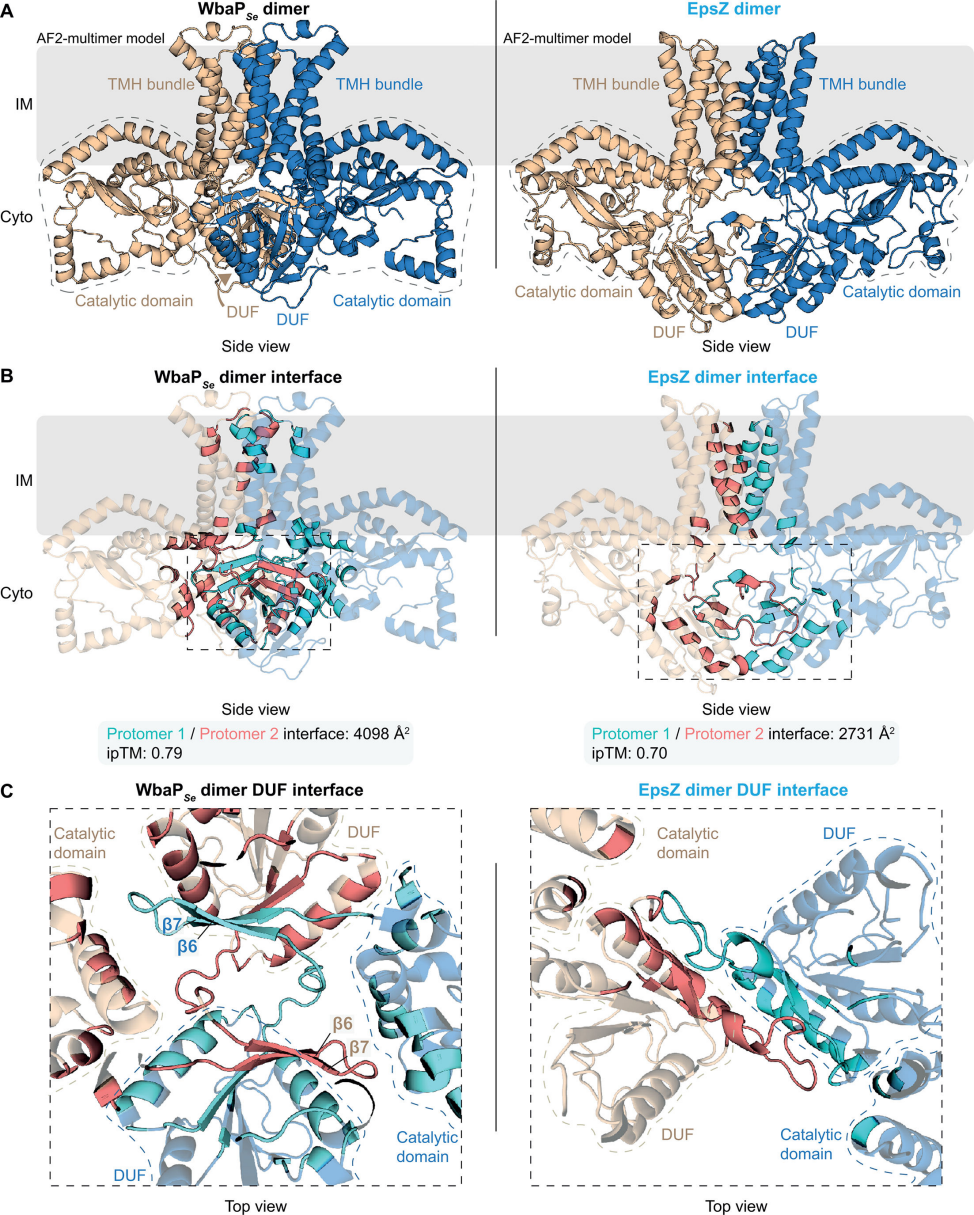
EpsZ alone may be compromised in dimer formation. (**A**) Computational structural comparison of WbaP*_Se_* and EpsZ dimers. Left and right panels, rank 1 AlphaFold2-Multimer models of dimeric WbaP*_Se_* and dimeric EpsZ. Individual protomers are colored blue and wheat. Regions with stippled outlines indicate the catalytic domains. The cytoplasmic DUFs and the TMH bundles are marked. The position of the proteins in the IM was computed using the PPM server (76). (**B**) Analysis of the dimer interface of the rank 1 AlphaFold2-Multimer models of the WbaP*_Se_* and EpsZ dimers. In both panels, residues at the interface between protomers are highlighted in cyan (contributed by the blue-colored protomer) and salmon (contributed by the wheat-colored protomer). Below, the overall surface area of the interface between the protomers and the corresponding ipTM value is shown. In both panels, the stippled area marks the interface within the cytoplasmic DUFs of the two protomers. The interface analysis was performed via PDBePISA (81). (**C**) Top view of zoomed-in image of the interface between the cytoplasmic DUFs of the protomers in the WbaP*_Se_* and EpsZ dimers. The zoomed region is marked by a stippled box in panel **B**. Both panels, residues at the dimer interface are highlighted as described in panel **A**, and stippled lines indicate the catalytic domains and DUFs of each protomer. Left panel, the characteristic β6/β7-hairpins involved in β-sheet augmentation are marked.

In the second step of the structural modeling, we considered that recent structural characterization and computational modeling suggest that WbaP*_Se_* forms a homodimer (14, 15), stabilized by the two cytoplasmic DUFs via the C-terminal β-hairpins (14). The structure of the WbaP*_Se_* homodimer has been solved at ~4.1 Å resolution (14). Therefore, we generated and compared AlphaFold2-Multimer models of WbaP*_Se_* and EpsZ dimers. WbaP*_Se_* was modeled as a homodimer (Fig. 4A) with high confidence and low predicted alignment error (pAE) (Fig. S8A and B). The predicted interface template modeling score (ipTM) of 0.79 is close to the stringent 0.80 cutoff (80), supporting an overall accurate prediction of the dimer interface (Fig. 4B). PDBePISA (81) analysis calculated a dimer interface area of 4,098 Å^2^ (Fig. 4B). As reported earlier (14), we observed that the two cytoplasmic DUFs contribute significantly to the dimer interface, with the β-hairpin of one protomer’s DUF participating in β-sheet augmentation (82) with the other protomer’s β-sheet and vice versa (Fig. 4C). Moreover, the tip of one protomer’s β-hairpin is close to the other protomer’s catalytic domain and vice versa (Fig. 4C). Additionally, the two protomers interface in a region involving the TMH bundles (Fig. 4B).

The model of the EpsZ homodimer also displayed a dimeric arrangement with the TMH bundles and the cytoplasmic DUFs in proximity (Fig. 4A); however, the PDBePISA analysis calculated a reduced dimer interface (2,731 Å^2^) compared to the WbaP*_Se_* dimer (4,098 Å^2^) (Fig. 4B), and no β-sheet augmentation in the DUFs’ interface (Fig. 4C). Moreover, the central part of the dimer, particularly the interface between the two DUFs, was only modeled with moderate confidence based on predicted local distance difference test (pLDDT) (Fig. S8A and C). Additionally, the pAE for the EpsZ dimer was higher compared to the WbaP*_Se_* dimer, indicating reduced confidence in the model’s spatial accuracy (Fig. S8C, compare to Fig. S8B). Finally, the ipTM value for the EpsZ dimer was 0.70, indicating a less confident interface prediction than for the WbaP*_Se_* dimer. Together, we conclude that the absence of the β-hairpin and the inability to model the EpsZ dimer with high confidence indicate that EpsZ alone cannot form a stable dimer.

### EpsW may facilitate the active, dimeric conformation of EpsZ

**Fig 5:**
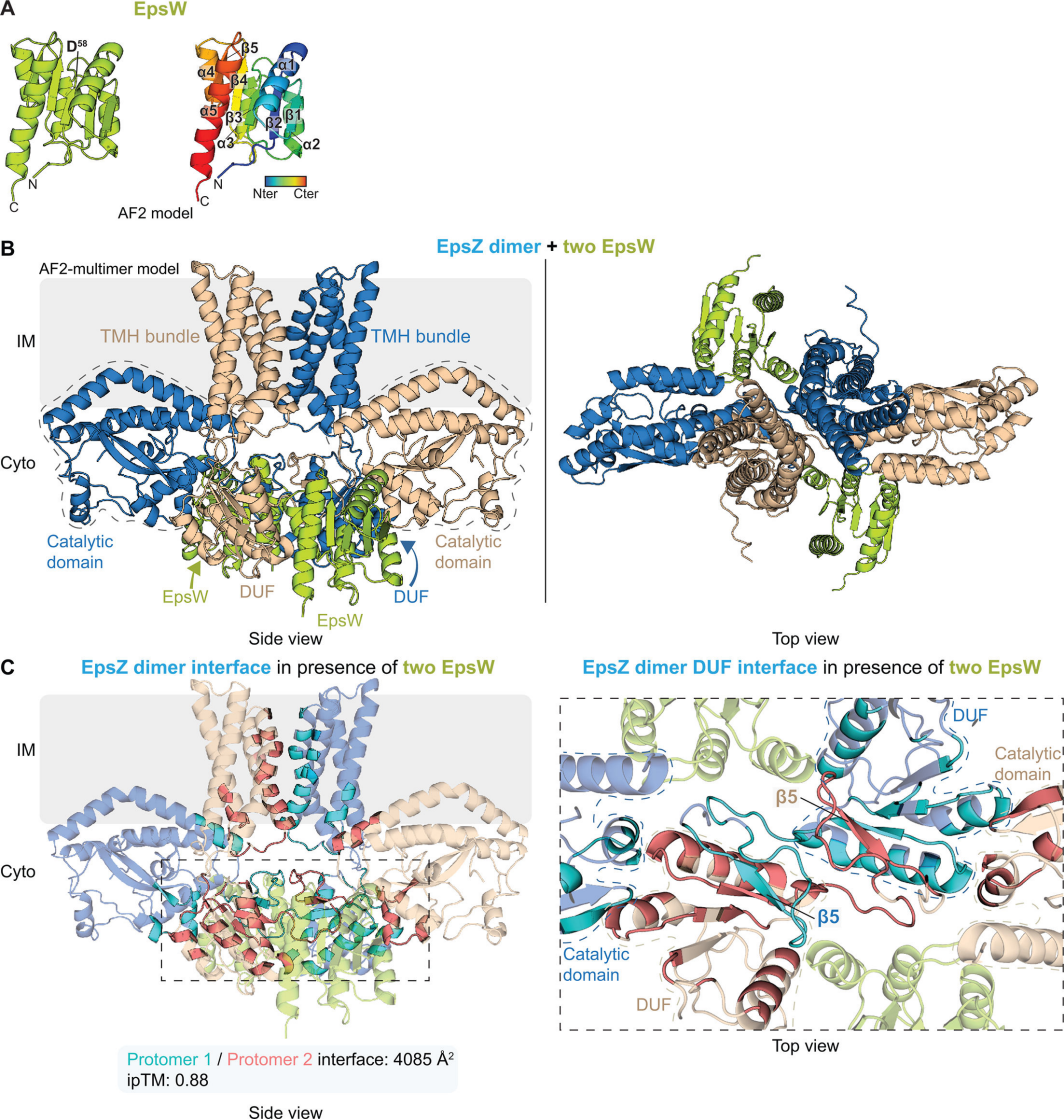
EpsW may facilitate the active, dimeric conformation of EpsZ. (**A**) Rank 1 AlphaFold2 model of EpsW. Left panel, EpsW in lime-green. The phosphorylated D^58^ based on reference 54 is indicated. Right panel, protein depicted in a gradient from blue (N-terminus) to red (C-terminus). Numbering of α-helices and β-strands is included. (**B**) Computational AlphaFold2-Multimer structural model of the EpsW-EpsZ heterocomplex in 2:2 stoichiometry. EpsW is colored lime-green, and EpsZ protomers are in blue and wheat. Model rank 1 is shown. Regions with stippled outlines indicate the catalytic domains; the cytoplasmic DUFs and the TMH bundles are marked. (**C**) Analysis of the EpsZ dimer interface within the AlphaFold2-Multimer model of the EpsW-EpsZ heterocomplex. Left panel, residues at the interface between the EpsZ protomers are highlighted in cyan (contributed by the blue-colored protomer) and salmon (contributed by the wheat-colored protomer). Below, the overall surface area of the interface between the EpsZ protomers and the corresponding ipTM value is shown. Right panel, zoomed-in top view of the interface between the cytoplasmic DUFs of the EpsZ protomers in the EpsW-EpsZ heterocomplex. The residues at the dimer interface are highlighted as in the left panel, and stippled lines indicate the catalytic domains and DUFs of each protomer. EpsW is colored lime-green. The β-sheet augmentation of the β5-strands is marked.

Finally, in the third step of the structural modeling, we explored an interaction between EpsZ and EpsW. To this end, we first generated a model of EpsW using AlphaFold2. Notably, it is not possible to model post-translational modifications (e.g., phosphorylation of D^58^ in EpsW that is phosphorylated by DifE [54]) using AlphaFold2 (83). Additionally, introducing substitutions in the sequence used for model generation (i.e., that could mimic phosphorylation) only has a limited impact on models compared to using the native sequences (84, 85). Thus, while EpsW~P is required to activate EPS biosynthesis in *M. xanthus* (54) and is the form of EpsW that interacts with EpsZ based on the proximity labeling experiments (Fig. 2A through C), we used the native EpsW sequence for modeling. In the high-confidence model of native EpsW (Fig. S8D and E), the EpsW monomer adopts the characteristic structure of the receiver domain of response regulators (86), consisting of a central five-stranded parallel β-sheet surrounded by five α-helices (Fig. 5A). A structure homology-based search using Foldseek (87) identified the BeF_3_^-^-activated form of the single-domain response regulator CheY*_E. coli_,* which mimics the phosphorylated form of CheY*_E. coli_*, in complex with CheZ (PDB: 1KMI [88]), with high confidence as the closest structural homolog of EpsW in the PDB^100^ database (probability, 1.0; sequence identity, 29.4%; and e-value, 7.14^−12^), suggesting that the EpsW model represents the activated phosphorylated state of the molecule.

Next, we generated a model comprising two copies of EpsZ and two copies of native EpsW using AlphaFold2-Multimer. In the EpsZ-EpsW heterocomplex, the two EpsW copies were modeled symmetrically on either side of the cytoplasmic part of the EpsZ dimer (Fig. 5B). The EpsZ-EpsW model was of high confidence, and with a low pAE and high ipTM score of 0.88 (Fig. S8F and G; Fig. 5C), indicating a highly confident prediction of the interfaces within the heterocomplex (80). The modeled structure of EpsW alone is similar to that of EpsW in complex with EpsZ (RMSD: 0.251 Å, C_α_ 831), suggesting that EpsW in this model is also in its activated, phosphorylated state.

In the EpsZ-EpsW heterocomplex, the EpsZ-EpsZ interface was located between the two DUFs and the TMH bundles (Fig. 5C), and the catalytic domains of the EpsZ protomers were swapped relative to the TMH bundles (Fig. 5B and C) when compared to the EpsZ dimer without EpsW (Fig. 4A and B). This resulted in an increased interface area between the EpsZ protomers to 4,085 Å^2^ (Fig. 5C). Moreover, we observed β-sheet augmentation within the EpsZ-EpsZ interface between the two DUFs (Fig. 5C). Specifically, the β5-strand of one protomer’s DUF (Fig. 3C) folds into the β-sheet of the adjacent protomer’s DUF and vice versa (Fig. 5C). We note that this interaction is different from that in the WbaP*_Se_* dimer, where the DUFs’ β-hairpins mediate β-sheet augmentation, rather than a strand of the central β-sheet (Fig. 4C). Importantly, EpsW does not contribute to the active site in EpsZ.

Together, our results from structural modeling predict a heterocomplex of two copies each of EpsZ and EpsW, in which EpsW directly interacts with EpsZ. Because EpsW is likely modeled in its phosphorylated state and does not contribute to the EpsZ active site, the structural modeling also strongly suggests that EpsW~P is a direct allosteric activator of EpsZ that facilitates the dimeric, active conformation of EpsZ, thereby stimulating Gal-1-P transferase activity and initiation of EPS biosynthesis.

### The EpsZ-EpsW mechanism may be conserved in orthologous Wzx/Wzy-dependent pathways

**Fig 6:**
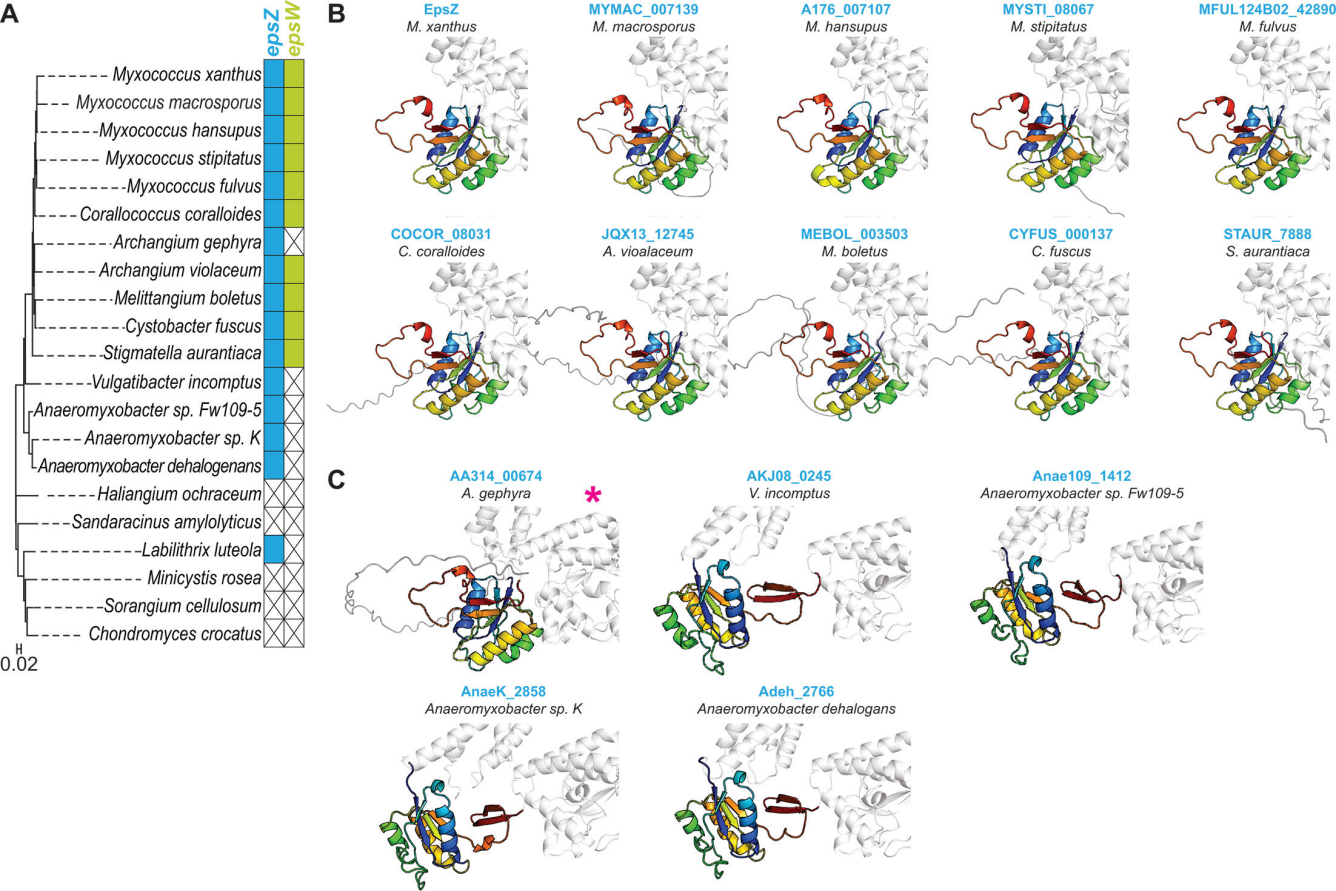
The absence of the β-hairpin in myxobacterial EpsZ orthologs largely correlates with the presence of an EpsW ortholog. (**A**) Left panel, phylogenetic tree of myxobacteria with fully sequenced genomes based on 16S rRNA sequences. Right panels, co-occurrence of EpsZ and EpsZ orthologs (blue) and EpsW and EpsW orthologs (lime-green) in myxobacteria as identified in references 23, 46. The absence of orthologs is indicated by an X. (**B**) Computational structural comparison of the cytoplasmic DUFs of EpsZ and the nine myxobacterial EpsZ orthologs co-occurring with an EpsW ortholog. In all panels, the cytoplasmic DUF of the rank 1 AlphaFold2 models of the full-length proteins is highlighted using a gradient from blue (N-terminus) to red (C-terminus). (**C**) Computational structural comparison of the cytoplasmic DUFs of myxobacterial EpsZ orthologs not co-occurring with an EpsW ortholog. In all panels, the cytoplasmic DUF of the rank 1 AlphaFold2 model is shown. The DUF is shown as in panel **B**. * indicates that the *A. gephyra* EpsZ ortholog does not have a β-hairpin.

To investigate whether the proposed EpsZ-EpsW mechanism is widespread, we searched for orthologous Wzx/Wzy-dependent EPS biosynthesis pathways in the 21 fully sequenced myxobacterial genomes (Table S7). We identified 15 EpsZ orthologs (23, 46), of which 9 co-occurred with an EpsW ortholog (Fig. 6A). Next, we performed structural modeling in the same three steps as for EpsZ/EpsW.

The models of the monomeric structures of the EpsZ orthologs were, generally, of high confidence (Fig. S9 and S10A). Only the *Labilitrix luteola* EpsZ ortholog was modeled with low confidence and has a DUF with an unusual structure comprising additional α-helices and β-strands (Fig. S10B); therefore, this protein was excluded from further analyses. Comparing the cytoplasmic DUFs of the remaining protein models, we found that this domain lacked the β-hairpin in 10 of the EpsZ orthologs and instead had an unstructured region connecting two of the β-sheet’s strands (Fig. 6B and C). The β-hairpin was present in four EpsZ orthologs (Fig. 6C). The EpsZ orthologs without the β-hairpin co-occurred with EpsW orthologs, except for *Archangium gephyra*, whereas EpsZ orthologs with the β-hairpin did not co-occur with EpsW orthologs (Fig. 6A through C). Thus, the absence or presence of the β-hairpin in these EpsZ orthologs almost perfectly correlates with the presence or absence of an EpsW ortholog.

**Fig 7:**
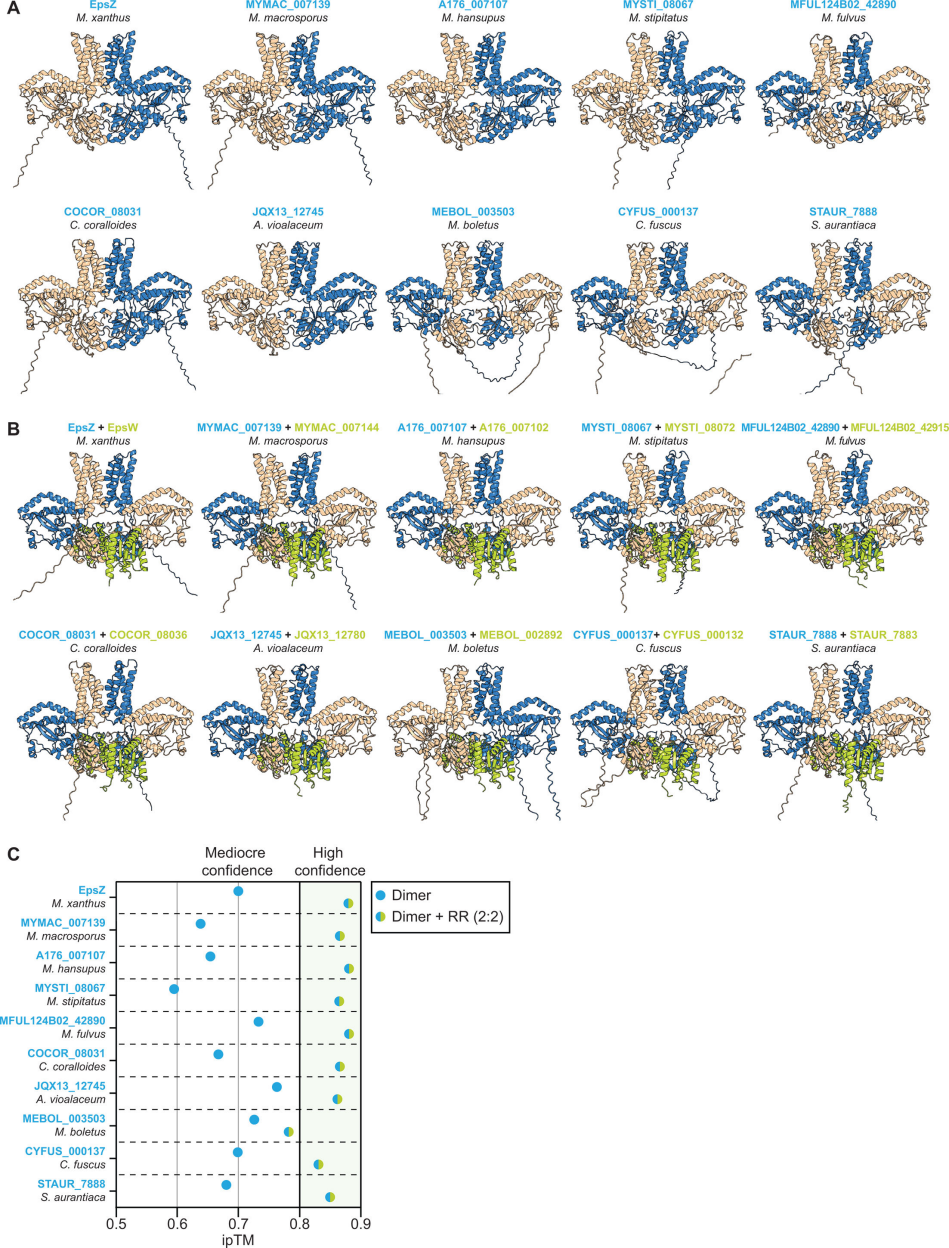
The EpsW/EpsZ mechanism may be conserved in myxobacteria. (**A**) Computational structural comparison of dimers of EpsZ and the nine myxobacterial EpsZ orthologs co-occurring with an EpsW ortholog. In all panels, the rank 1 AlphaFold2-Multimer model of the dimeric protein is shown, and the individual protomers are colored blue and wheat. (**B**) Computational structural comparison of dimers EpsZ and nine myxobacterial EpsZ orthologs together with the corresponding EpsW ortholog in 2:2 stoichiometry. In all panels, rank 1 AlphaFold2-Multimer model of the heterocomplex is shown; the individual EpsZ ortholog protomers are colored blue and wheat, and the two copies of the EpsW ortholog in lime-green. (**C**) ipTM values of the rank 1 AlphaFold2-Multimer models in panels **A** and **B**. *X*-axis, data points represent the ipTM values of the rank 1 model of the dimer of EpsZ and the EpsZ orthologs, either without (points colored blue) or with the EpsW ortholog (points colored blue/lime-green). An ipTM score above 0.80 indicates a confident interface prediction, scores between 0.60 and 0.80 fall into a gray zone where predictions may or may not be correct, and scores below 0.60 indicate a failed prediction (80).

Dimeric models of the nine EpsZ orthologs that co-occurred with an EpsW ortholog (Fig. 7A) were overall of mediocre confidence (Fig. S11). In five of the nine dimers, the domain arrangement resembled that of the EpsZ dimer, but in the remaining four models, the catalytic domains were swapped relative to the TMH bundles (Fig. 7A). Finally, we modeled the nine heterocomplexes containing two copies of the EpsZ ortholog and two copies of the corresponding EpsW ortholog. In these nine models, the two copies of the EpsW orthologs were positioned symmetrically on either side of the cytoplasmic part of the dimer of the EpsZ orthologs, reminiscent of the heterocomplex in *M. xanthus* (Fig. 7B). Except for the *Melittangium boletus* model, the models of the heterocomplexes had high ipTM scores and low pAE values (Fig. 7C; Fig. S12), indicating high confidence in the predicted interactions in the heterocomplexes. Moreover, the ipTM scores and pAE values of the heterocomplexes were markedly higher than those of the corresponding homodimeric complexes (Fig. 7C; Fig. S11 and S12).

Collectively, these computational analyses suggest that the direct interaction of a single-domain response regulator (i.e., EpsW and its orthologs) and a large monoPGT lacking the characteristic β-hairpin (i.e., EpsZ and its orthologs) is conserved in several other myxobacteria, suggesting that the EpsW/EpsZ mechanism for activating a large monoPGT is widespread.

## DISCUSSION

In this study, we analyzed how the single-domain response regulator EpsW, upon phosphorylation by DifE, activates EPS biosynthesis. Three key observations support a model in which EpsW~P activates EPS biosynthesis by stimulating EpsZ Gal-1-P transferase activity. First, global transcriptomic and proteomic analyses indicate that EpsW~P stimulates EPS biosynthesis at the post-translational level. Second, mTurbo-based proximity labeling in *M. xanthus* reveals that EpsW~P is in close proximity to and likely directly interacts with EpsZ. Third, *in vivo* heterologous expression experiments in *S. enterica* show that the low level of Gal-1-P transferase activity of EpsZ alone is significantly enhanced by co-expressing EpsW.

Computational structural biology analyses suggest that EpsZ, similar to other large monoPGTs, has the C-terminal canonical catalytic domain partially embedded in the cytoplasmic leaflet of the IM, an N-terminal TMH bundle, and a central cytoplasmic DUF. However, the cytoplasmic DUF of EpsZ lacks the characteristic β-hairpin that contributes most of the dimer interface in the homodimeric WbaP*_Se_* and stabilizes the dimer by β-sheet augmentation (14). This difference was reflected in the lower confidence of the EpsZ homodimer model and the smaller interface area compared to dimeric WbaP*_Se_*. By contrast, the model of the EpsZ-EpsW heterocomplex with 2:2 stoichiometry was of high confidence. In this complex, EpsZ had a dimeric arrangement, and each of the two EpsW copies symmetrically and directly contacts an EpsZ protomer’s DUF and catalytic domain on both sides of the EpsZ dimer. In the EpsZ-EpsW heterocomplex, the two catalytic domains of EpsZ were swapped relative to the two TMH bundles. Moreover, the EpsZ dimer interface area in the heterocomplex configuration increased to a similar level as in the WbaP*_Se_* dimer. Based on these models, we posit that EpsZ alone cannot efficiently dimerize and that EpsW~P is an allosteric regulator, which facilitates the formation of the dimeric conformation of EpsZ upon direct interaction. Because EpsW~P activates EpsZ Gal-1-P transferase activity, we suggest that this dimeric conformation, analogous to the functional WbaP*_Se_* homodimer, represents the active state of EpsZ. Similar inferences can be drawn for myxobacterial EpsZ orthologs and their partner EpsW orthologs since structural modeling also supports, with high confidence, the formation of heterocomplexes reminiscent of the *M. xanthus* EpsZ/EpsW heterocomplex. Together, these findings support that a phosphorylated single-domain response regulator stimulates, by direct protein-protein interaction, the activity of a large monoPGT in several myxobacteria.

Post-translational modification of the *S. aureus* small monoPGT CapM by Tyr phosphorylation was suggested to stimulate enzymatic activity (39). Our results suggest a novel mode of post-translational regulation in which a single domain response regulator allosterically stimulates the initiating large monoPGT via direct protein-protein interaction. The EpsZ-EpsW mechanism adds to the list of mechanisms for post-translational regulation of the initial step of polysaccharide biosynthesis as described for synthase-dependent pathways (41–45). Because the accumulation of the Eps proteins is independent of EpsW, we speculate that the suggested mechanism provides *M. xanthus* with the ability to swiftly switch on the EPS pathway in response to T4P extension and other stimuli potentially sensed by the Dif chemosensory system. We also note that EPS biosynthesis is energy-costly and uses the lipid carrier Und-P, which is required for the biosynthesis of the essential peptidoglycan and other polysaccharides in *M. xanthus* (50). Thus, it is likely beneficial to regulate EPS biosynthesis at the initial step rather than in further downstream steps, in which repeat units would accumulate without being incorporated into the final EPS chain, and Und-P would be titrated away from other biosynthetic pathways.

Based on structural modeling, we suggest that stimulation of a large monoPGT by a phosphorylated single-domain response regulator domain is likely conserved across several myxobacteria, supporting that it could be more widespread in bacteria. Interestingly, a recent sequence-based bioinformatic analysis revealed complexity in the domain architecture of the small monoPGT family (13). These monoPGTs may incorporate additional domains, such as sugar-modifying and regulatory domains, including receiver domains of response regulators (13). However, because small monoPGTs are functional monomers (11), the regulation mechanism may not involve dimer formation. Yet, these observations indicate complex regulatory and functional diversity in the initial step of polysaccharide biosynthesis, supporting the idea that receiver domains could be involved in regulating monoPGTs in other organisms.

## MATERIALS AND METHODS

### Strains and cell growth

All *M. xanthus* strains used in this study are derivatives of the WT strain DK1622 (89) and are listed in Table S7. Plasmids and oligonucleotides are listed in Tables S8 and S9, respectively. In-frame deletions in *M. xanthus* were constructed via two-step homologous recombination as described (90). Plasmids for complementation experiments were integrated in a single copy by site-specific recombination at the Mx8 *attB* site or by homologous recombination at the *18-19* intergenic locus. All plasmids were verified by DNA sequencing, and all strains were verified by PCR. *M. xanthus* cultures were grown at 32°C in 1% CTT broth (1% [wt/vol] Bacto casitone, 10 mM Tris-HCl [pH 8.0], 1 mM K_2_HPO_4_/KH_2_PO_4_ [pH 7.6], and 8 mM MgSO_4_) or on 1.5% agar supplemented with 1% CTT and kanamycin (50 µg mL^−1^) or oxytetracycline (10 µg mL^−1^) when appropriate (91). Gene expression from the vanillate-inducible promoter (92) was induced with 7.5 µM vanillate. Plasmids were propagated in *Escherichia coli* NEB Turbo at 37°C in lysogeny broth (LB) (93) supplemented with kanamycin (50 µg mL^−1^) or tetracycline (20 µg mL^−1^) when required. *S. enterica* was cultured at 37°C in LB supplemented with ampicillin (100 µg mL^−1^), chloramphenicol (30 µg mL^−1^), and 0.2% or 0.4% (wt/vol) L-arabinose when appropriate.

### Plasmid construction

For pMP036 (for generating the in-frame deletion in *epsW*), up- and downstream fragments were amplified from genomic DNA of DK1622 using the primer pairs 7420-A/7420-B and 7420-C/7420-D, respectively. Subsequently, the AB and CD fragments were used as templates for an overlapping PCR with the primer pair 7420-A/7420-B to generate the AD fragment. The AD fragment was digested with KpnI/XbaI and cloned into pBJ114.

For pDJS102 (for generating the in-frame deletion in *difE*), up- and downstream fragments were amplified from genomic DNA of DK1622 using the primer pairs 6692-A/6692-B and 6692-C/6692-D, respectively. Subsequently, the AB and CD fragments were used as templates for an overlapping PCR with the primer pair 6692-A/6692-D to generate the AD fragment. The AD fragment was digested with EcoRI/XbaI and cloned into pBJ114.

For pJSc002 (for generating the in-frame deletion in *difD*), up- and downstream fragments were amplified from genomic DNA using the primer pairs 6693-A/6693-B and 6693-C/6693-D, respectively. Subsequently, the AB and CD fragments were used as templates for an overlapping PCR with the primer pair 6693-A/6693-D to generate the AD fragment. The AD fragment was digested with KpnI/XbaI and cloned into pBJ114.

For pJSc003 (for the generation of an in-frame deletion in *difG*), up- and downstream fragments were amplified from genomic DNA using the primer pairs 6691_A/6691_B and 6691_C/6691_D, respectively. Subsequently, the AB and CD fragments were used as templates for an overlapping PCR with the primer pair 6691_A/6691_D to generate the AD fragment. The AD fragment was digested with KpnI/XbaI and cloned into pBJ114.

For pMP145 (plasmid for the expression of P*_pilA_ epsW* from the Mx8 *attB* site), the *epsW* fragment was amplified with the primer pair 7420_PpilA fw /7420_PpilA rev from the genomic DNA of DK1622, digested with XbaI/HindIII, and cloned into pSW105.

For pJSc113 (for generation of a strain ectopically expressing *epsW* from the *pilA* promoter at the Mx8 *attB* site), a fragment was cleaved from pMP145 using EcoRI/HindIII. The resulting fragment was cloned into pSWU30.

For pJSc105 (for generation of a strain ectopically expressing *mTurbo-epsW-FLAG* from the *pilA* promoter at the Mx8 *attB* site), fragment one was generated from pMH97 (67) using mTurbo-P*_pilA_*-for and mTurbo-epsW-FLAG int rev. Fragment two was generated from genomic DNA using mTurbo-epsW-FLAG int for and EpsW-FLAG-rev. An overlap extension PCR using mTurbo-P*_pilA_*-for and EpsW-FLAG-rev was performed on fragments one and two. The resulting fragment was digested with XbaI/HindIII and cloned into pSW105.

For pJSc143 (plasmid for expression of *epsW-His*_10_ from the arabinose-inducible promoter), the *epsW-His*_10_ fragment was amplified with the primer pair 7420_PpilA fw/EpsW-His-rev-HindIII from genomic DNA of DK1622, digested with XbaI/HindIII, and cloned into pBAD33.

For pJSc146 (plasmid for expression of P*_pilA_ epsW-His*_10_ from the Mx8 *attB* site), the *epsW-His*_10_ fragment was amplified with the primer pair 7420_PpilA fw/EpsW-His-rev-HindIII from genomic DNA of DK1622, digested with XbaI/HindIII, and cloned into pSW105.

### Detection of EPS biosynthesis

Colony-based colorimetric EPS assays were performed as described (46). Briefly, cells grown exponentially in suspension cultures were sedimented by centrifugation (3 min, 6,000 × *g* at room temperature [RT]) and resuspended in 1% CTT to a calculated cell density of 7 × 10^9^ cells mL^−1^. Next, 20 µL of the cell suspension was spotted onto 0.5% agar plates containing 0.5% CTT supplemented with either 10 µg mL^−1^ Trypan blue or 20  µg mL^−1^ Congo red. Plates were incubated at 32°C and imaged after 24 h.

### Detection of LPS O-antigen

LPS was extracted from *S. enterica* as described (94, 95). Briefly, cells were grown and harvested as described for *S. enterica* immunoblots. To lyse cells, cell pellets were resuspended in 150 µL of lysis buffer (0.5 M of Tris-HCl [pH 6.8], 2% [wt/vol] SDS, and 4% β-mercaptoethanol [vol/vol]) and boiled for 10 min. Then, 10 µL of Proteinase K (10 mg mL^–1^) was added, and samples were incubated at 60°C overnight. To remove proteins, 150 µL of pre-warmed (70°C) 90% phenol solution was added, and extracts were incubated at 70°C for 15 min with vortexing every 5 min. Then, samples were incubated for 10 min on ice and centrifuged at 10,000 × *g* for 10 min. Finally, 50 µL of the clear aqueous phase was transferred to a clean microcentrifuge tube, and 3× loading buffer (0.187 M Tris-HCl [pH 6.8], 6% [wt/vol] SDS, 15% [vol/vol] β-mercaptoethanol, 30% [vol/vol] glycerol, and 0.03% [wt/vol] bromophenol blue) was added. LPS samples were separated on 14% (wt/vol) Tricine-SDS-PAGE and stained with silver nitrate (94). For immunoblot detection of *Salmonella* O-antigen, LPS samples were separated on 12% Mini-PROTEAN TGX Precast Protein Gels (BioRad), transferred to a nitrocellulose membrane, and probed with rabbit *Salmonella* O antiserum group B (Difco, Beckton Dickinson ref. number 229481) (1:250) together with IRDye 680CW goat α-rabbit immunoglobulin G (1:10,000) (LI-COR) as secondary antibody and detected with a LI-COR Odyssey infrared imaging system (LI-COR).

### Motility assays

Motility assays were conducted as described (96). Briefly, cells grown exponentially in suspension cultures were sedimented by centrifugation (3 min, 6,000 × *g*, RT) and resuspended in 1% CTT medium to a calculated cell density of 7 × 10⁹ cells mL^−1^. Next, 5 µL aliquots of this cell suspension were placed onto 0.5% agar supplemented with 0.5% CTT, followed by incubation at 32°C for 24 h. Imaging was performed using a M205FA stereomicroscope (Leica Microsystems) equipped with a Hamamatsu ORCA-Flash V2 digital CMOS camera (Hamamatsu Photonics).

### Immunoblot analysis

For *M. xanthus*, immunoblot analyses were performed as described (97). Samples were prepared by harvesting *M. xanthus* cells from exponentially growing suspension cultures, followed by resuspension of the cell pellet in SDS lysis buffer (60 mM Tris-HCl [pH 6.8], 2% SDS [wt/vol], 10% glycerol [vol/vol], 5 mM ethylenediaminetetraacetic acid, 0.1 M dithiothreitol, and 0.005% bromophenol blue [wt/vol]). Proteins from an equal number of cells were loaded per sample. Blots were probed with rabbit polyclonal α-FLAG (1:5,000; Proteintech), mouse monoclonal α-His_6_-tag (1:2,000, Proteintech), and rabbit α-PilC (1:2,000) (98) primary antibodies. Rabbit α-FLAG and α-PilC antibodies were used together with horseradish peroxidase (HRP)-conjugated α-rabbit immunoglobulin G (1:15,000; Sigma) as the secondary antibody. Mouse α-His_6_-tag antibodies were used together with HRP-conjugated α-mouse immunoglobulin G (1:2,000; GE Healthcare) as the secondary antibody. For detecting biotinylated proteins, blots were probed with Strep-Tactin-HRP conjugate (1:5,000; IBA Lifesciences). Blots were developed using Immobilon Forte Western HRP substrate (Millipore) and imaged using the luminescent imager analyzer LAS-4000 (Fujifilm).

For *S. enterica,* cells were grown at 37°C overnight on LB plates supplemented with antibiotics and 0.0%, 0.2%, or 0.4% arabinose. Biomass was collected from each plate, resuspended in 5 mL phosphate-buffered saline (PBS) (137 mM NaCl, 2.7 mM KCl, 10 mM Na_2_HPO_4_, and 1.8 mM KH_2_PO_4_ [pH 7.2]), and the optical density at 600 nm was adjusted to 5. One milliliter of normalized suspensions was transferred to a microcentrifuge tube, and cells were harvested by centrifugation (8,000 × *g*, 3 min, RT). Cells were resuspended in 1× Laemmli sample buffer (69.4 mM Tris-HCl [pH 6.8], 1.1% [wt/vol] SDS, 355 mM β-mercaptoethanol, 11.1% glycerol [vol/vol], and 0.005% bromophenol blue [wt/vol]) to generate whole cell lysates. Samples were incubated for 5 min at 65°C, and proteins from an equal number of cells were loaded per sample. Proteins were separated by SDS-PAGE on 12% Mini-PROTEAN TGX Precast Protein Gels (Bio-Rad), transferred to a nitrocellulose membrane, and blots were probed with mouse α-FLAG M2 (Sigma) (1:10,000) and α-DnaK antibodies (Novus Biologicals) (1:5,000) primary antibodies, which were detected using IRDye 680CW-conjugated goat α-mouse IgG (H + L) (LI-COR) and IRDye 800CW-conjugated goat α-mouse IgM, respectively, and imaged using a LI-COR Odyssey infrared imaging system.

### RNA-seq analysis

Total RNA from exponentially growing *M. xanthus* cells in 20 mL suspension culture was extracted using the Monarch Total RNA Miniprep Kit (New England Biolabs) according to the manufacturer’s protocol. Next, Turbo DNase (Thermo Fisher Scientific) was added to the RNA following the manufacturer’s protocol and subsequently removed using the Monarch RNA Cleanup Kit (50 µg; New England Biolabs). Analysis of RNA integrity, ribosomal RNA (rRNA) depletion, library preparation, and sequencing were performed by Vertis Biotechnologie. For all samples, RNA integrity was analyzed by capillary electrophoresis using the MultiNA microchip electrophoresis system (Shimadzu). For the removal of rRNA, an in-house protocol was used. Briefly, a mixture of *in vitro*-transcribed biotin-labeled RNA probes directed against 5S, 16S, and 23S bacterial rRNA was mixed with the total RNA samples. The hybridized rRNA molecules were then removed using streptavidin magnetic beads. Next, ribodepleted-RNA samples were fragmented using ultrasound (two pulses of 30 s each at 4°C). Then, an oligonucleotide adapter was ligated to the 3′ end of the RNA molecules. First-strand cDNA synthesis was performed using M-MLV reverse transcriptase and the 3′ adapter as primer. The first-strand cDNA was purified, and the 5′ Illumina TruSeq sequencing adapter was ligated to the 3′ end of the antisense cDNA. The resulting cDNA was PCR-amplified to about 10–20 ng µL^−1^ using a high-fidelity DNA polymerase (14 PCR cycles). The cDNA was purified using an Agencourt AMPure XP kit (Beckman Coulter Genomics). For Illumina NextSeq sequencing, the samples were pooled in approximately equimolar amounts. The cDNA pool was purified using the Agencourt AMPure XP kit (Beckman Coulter Genomics). At all steps, quality was assessed via the MultiNA Microchip Electrophoresis System (Shimadzu). The cDNA pool was sequenced on an Illumina NextSeq 500 system using 1 × 75 bp read length. All samples had between ~ 15 and ~20 Mio reads.

The differential gene expression analysis was performed using the RNA-seq pipeline Curare 0.4.4 (99). The reads were preprocessed with Trim Galore (100), trimming low-quality ends (<20) and discarding reads smaller than 50 bp. Preprocessed reads were then aligned using Bowtie2 2.4.5 in “very-sensitive” mode and with “–mm” option (101). The resulting alignment files were processed with SAMtools 1.15.1 (102). The subsequent assignment of mapped reads to genome features was done using the featureCounts of the subread 2.0.1 package (103). featureCounts was run with “-s 1” settings, assigning reads in a strand-specific manner to the “CDS” features. The differential gene expression was determined with DESeq2 1.34.0 (61). The genome and annotation of *M. xanthus* DK1622 (ASM1268v1) were used for all analyses.

The count table and mapping results have been deposited at EBI ArrayExpress under accession number E-MTAB-14794.

### Proteomic analysis using data-independent acquisition-mass spectrometry

Whole-cell proteomics experiments were performed based on reference 23. Briefly, *M. xanthus* cells from 10 mL of an exponentially growing suspension culture were harvested by centrifugation (3 min, 10,000* g*, RT) and washed twice in 0.5 mL PBS (137 mM NaCl, 2.7 mM KCl, 10 mM Na_2_HPO_4_, and 1.8 mM KH_2_PO_4_ [pH 7.5] supplemented with 2× protease inhibitor (Roche). The cells were harvested by centrifugation and resuspended in 0.2 mL of 0.1 M ammonium bicarbonate containing 2% (wt/vol) sodium lauroyl sarcosinate (SLS) and incubated at 95°C for 1 h. Next, the samples were centrifuged at 14,000 × *g* for 5 min, and the supernatant was harvested. Next, 1.2 mL freezer-cold acetone was added to the supernatant, mixed, and incubated at −80°C for at least 2 h. Samples were centrifuged at 21,000 × *g* for 15 min at 4°C, and the supernatant was discarded. The pellet was washed thrice with freezer-cold methanol, dried, and the methanol was completely removed. The precipitated protein pellets were solubilized using 200 µL of 0.5% SLS and heat exposure. The protein amount was determined by bicinchoninic acid (BCA) assay (Thermo Fisher Scientific). Next, proteins were reduced using 5 mM Tris (2-carboxyethyl) phosphine (TCEP, Thermo Fisher Scientific) at 90°C for 15 min and alkylated with 10 mM iodoacetamide (Sigma Aldrich) at 25°C for 30 min in the dark. Fifty micrograms of protein was digested using 1 µg trypsin (Serva) at 30°C overnight.

*M. xanthus* peptide samples were acidified to precipitate and remove SLS. Next, the peptides were desalted using C18 solid phase extraction cartridges (Macherey-Nagel), which were prepared for loading the peptide sample by first adding acetonitrile (ACN), followed by 0.1% trifluoroacetic acid (TFA, Thermo Fisher Scientific). The cartridges were equilibrated, and peptides were loaded and washed with buffer containing 5% ACN/0.1% TFA. Peptides were eluted with 50% ACN and 0.1% TFA. Dried peptides were reconstituted in 0.1% TFA and then analyzed using liquid chromatography-mass spectrometry (LC-MS) executed on an Ultimate 3000 RSLCnano connected to an Exploris 480 Mass Spectrometer (all Thermo Fisher Scientific) and a HPLC C18 column packed in-house (75 µm × 42 cm). *M. xanthus* peptides were separated using the following gradient: 94% solvent A (0.15% formic acid) and 6% solvent B (99.85% acetonitrile and 0.15% formic acid) to 25% solvent B over 95 min at a flow rate of 300 nL min^−1^, followed by an additional increase of solvent B to 35% over 25 min.

MS raw data were acquired in data-independent acquisition (DIA) mode. Briefly, the spray voltage was set to 2.3 kV, the funnel radio frequency level was set to 40, and the ion transfer capillary was heated to 275°C. For DIA experiments, full MS resolutions were set to 120,000 at *m/z* 200 and full MS, with the AGC (automatic gain control) target set to 300% and ion accumulation time of 50 ms. The AGC target value for fragment spectra was set to 3,000%. Forty-five windows, each set to 14 Da plus 1 Da overlap, were used. The resolution was set to 15,000 with MS/MS IT to 22 ms. Stepped high-energy collision dissociation (HCD) collision energies of 25%, 27.5%, and 30% were applied. MS1 data were acquired in profile mode, and MS2 DIA data in centroid mode.

DIA data analysis was performed using the neural network-based DIA-NN suite version 1.8 (104) in combination with an *M. xanthus* protein database from UniProt. A data set-centric spectral library was generated for the DIA analysis. DIA-NN applied noise interference correction (mass correction, RT prediction, and precursor/fragment co-elution correlation) and peptide precursor signal extraction from the raw DIA-NN data. The following settings were used for the analysis: full tryptic digestion with two missed cleavage sites was allowed, with oxidized methionines and carbamidomethylated cysteines as modifications. “Match between runs” and “remove likely interferences” were enabled. The NN classifier was set to the single-pass mode, and protein inference was based on genes. Quantification was performed using the “any LC (high accuracy)” strategy, with cross-run normalization set to RT-dependent. Library generation was set to smart profiling. Outputs from DIA-NN were further evaluated using a SafeQuant (105, 106) script modified to process DIA-NN results. For global analyses, imputation of missing values was performed using a normal distribution function, similar to the strategy implemented in the Perseus statistical software package (107).

The mass spectrometry proteomics data of whole cell proteomics experiments have been deposited to the ProteomeXchange Consortium (108) via the PRIDE (109) partner repository with the data set identifier PXD063981.

### Proximity labeling experiments

Protein interactor identification using proximity labeling and shotgun proteomics was performed as described (67). Briefly, 25 µM biotin was added to exponentially growing *M. xanthus* strains expressing mTurbo-fused proteins in suspension cultures, and growth was allowed to proceed for 3 h. Cells were harvested at 8,000 × *g* for 5 min at RT and washed thrice in 5 mL of TPM (10 mM Tris-HCl [pH 7.6], 1 mM potassium phosphate [pH 7.6], and 8 mM MgSO_4_). The cells were then lysed in 0.6 mL RIPA buffer (50 mM Tris-HCl [pH 7], 150 mM NaCl, 1% Triton X-100 [wt/vol], 0.5% sodium deoxycholate [wt/vol], and 0.2% SDS [wt/vol]) also containing 2× protease inhibitor cocktail (Roche) and 5 µL benzonase (Merck). Intact cells were sedimented, and the supernatant was applied to equilibrated G25 desalting columns (GE Healthcare). Next, the protein concentration in the desalted lysate was measured using the BCA assay (Thermo Fisher Scientific), and protein concentration was adjusted to 2 mg mL^−1^ in all samples. A volume of 50 µL of equilibrated magnetic Pierce Streptavidin beads (Thermo Fisher Scientific) was added to the desalted lysate, and the mixture was incubated for 1 h at 4°C with mild shaking. Next, the beads were harvested and washed twice in RIPA, twice in 1 M KCl, and thrice in 50 mM Tris-HCl (pH 7.6). Proteins were digested using an on-bead digest with 1 µg trypsin overnight at 30°C in the presence of 1 mM TCEP. Following digestion, the peptides were further alkylated with 2 mM iodoacetamide, and peptides were purified using solid-phase extraction on Chromabond spin columns (Macherey-Nagel). Peptides were then loaded on the LC-MS system described above. Peptide separation was carried out using a constant flow rate of 300 nL min^−1^ and a 45-min gradient from 6% to 35% LC buffer B (99.85% acetonitrile/0.15% formic acid). Eluting peptides were ionized at 2.3 kV. MS raw data were acquired in data-dependent mode with a MS1 resolution of 60,000 full width at half maximum (at *m/z* 200), followed by MS/MS scans of the most intense ions within 1 s (cycle 1 s). The dynamic exclusion time was set to 14 s, and the ion accumulation time was set to 50 ms for MS1 and 50 ms at 17,500 resolution for MS/MS scans. The AGC was set to 3 × 10^6^ for MS survey scans and 2 × 10^5^ for MS/MS scans. The quadrupole isolation was 1.5 *m/z*, and the collision was induced with an HCD collision energy of 27%.

MS raw data were analyzed with MaxQuant (110) and an *M. xanthus* UniProt database. MaxQuant was used with standard settings without the “match between runs” option. The MaxQuant “proteinGroups.txt” output file was further processed by the SafeQuant R package for statistical analysis (105, 106). The same data imputation method was used for the global proteome analysis. The criteria for significant enrichment in samples over that of the control were an abundance difference of ≥8 (log_2_-fold enrichment of ≥3.0) and a *P*-value of ≤0.0001 (−log_10_ *P*-value ≥ 4.0). *P*-values were calculated using an eBayes method provided by the Limma software package (111).

Proximity labeling experiment data have been deposited to the ProteomeXchange Consortium (108) via the PRIDE (109) partner repository with the data set identifier PXD063973.

### Bioinformatics

Gene and protein sequences were fetched from the KEGG (112) and UniProt (113) databases. The operon structure of the *eps* loci was generated using the RNA-seq and cappable-seq data from reference 60. The phylogenetic tree of Myxobacteria was generated in MEGA-X (114) using the neighbor-joining method (115) and the genome sequences listed in Table S10. Myxobacterial EpsZ and EpsW orthologs were previously identified (23, 46).

Protein structure predictions were conducted using AlphaFold2 and AlphaFold2-Multimer_v3 implemented via ColabFold (116–118) with the Alphafold2_mmseqs2 notebook and default settings. To assess the quality of AlphaFold-generated models, pLDDT and pAE graphs of five models were generated using a custom-made Matlab R2020a (The MathWorks) script. These models were assessed based on a combination of pLDDT, pAE values, and the ipTM scores. The best-ranked models were then selected for analysis and presentation. Per-residue model confidence was inferred from pLDDT values (>90, high accuracy; 70–90, generally good accuracy; 50–70, low accuracy; <50, should not be interpreted) (116). To analyze the relative positioning of residues, the pAE, measured in angstroms, was assessed. The lower the pAE value, the higher the accuracy of the relative position of residue pairs and, consequently, the relative position of domains/subunits/proteins (116). ipTM values (117) were further used to assess the reliability of interfaces in multimeric models. An ipTM score above 0.80 indicates an accurate interface, scores between 0.60 and 0.80 fall into a gray zone where predictions may or may not be correct, and scores below 0.60 indicate a failed prediction (80, 119). Protein interfaces in multimeric structural models were determined using PDBePISA with default settings (81).

To inspect and visualize structural models, we used PyMOL (The PyMOL Molecular Graphics System, version 2.4.1, Schrödinger, LLC). Models colored based on pLDDT values were made using a custom command line (spectrum b, red_yellow_green_cyan_blue, minimum = 50, maximum = 90). Foldseek (87) was used to identify protein homologs in the PDB^100^ database. The positioning of protein structural models within the IM was assessed using the PPM 3.0 web server (76) with default settings and membrane type set to “Gram-negative bacteria inner membrane.” The coordinates of all structural models generated in this study have been deposited in the Edmond research data repository under reference 120.

### Statistical analysis

Data shown for EPS assays, T4P-dependent motility, and immunoblots were obtained in three independent experiments with similar results.

## Data Availability

The data supporting the findings of this study are all included in the manuscript and its supplemental file. The count table and mapping results of the RNA-seq experiment have been deposited at EBI ArrayExpress under accession number E-MTAB-14794. The mass spectrometry data of whole cell proteomics and proximity labeling experiments have been deposited to the ProteomeXchange Consortium via the PRIDE partner repository with the data set identifiers PXD063981 and PXD063973, respectively. All materials are available from the corresponding author.

## SUPPLEMENTAL MATERIAL

Supplemental Information mbio.02986-25-s0001.pdf Figures S1 to S12 and Tables S1 to S10.

ASM does not own the copyrights to Supplemental Material that may be linked to, or accessed through, an article. The authors have granted ASM a non-exclusive, world-wide license to publish the Supplemental Material files. Please contact the corresponding author directly for reuse.
