# Supplementary material for: A single-domain response regulator activates exopolysaccharide biosynthesis by interaction with the initiating phosphoglycosyl transferase
Source: mBio. 2025 Nov 28;17(1):e02986-25. doi: 10.1128/mbio.02986-25 (PMC12802240; doi:10.1128/mbio.02986-25)
Supplement: Supplemental Information — Figures S1 to S12 and Tables S1 to S10. [file mbio.02986-25-s0001.pdf]

Supporting Information

A single-domain response regulator activates exopolysaccharide biosynthesis by interaction with the initiating phosphoglycosyl transferase

Johannes Schwabe, Julia Monjaras-Feria, Timo Glatter, Patrick Blumenkamp, Oliver Rupp, Alexander Goesmann, Miguel A. Valvano and Lotte Søgaard-Andersen

This file contains:

- Supplementary Figures 1-12 incl. Legends
- Supplementary Tables 1-10
- Supplementary References

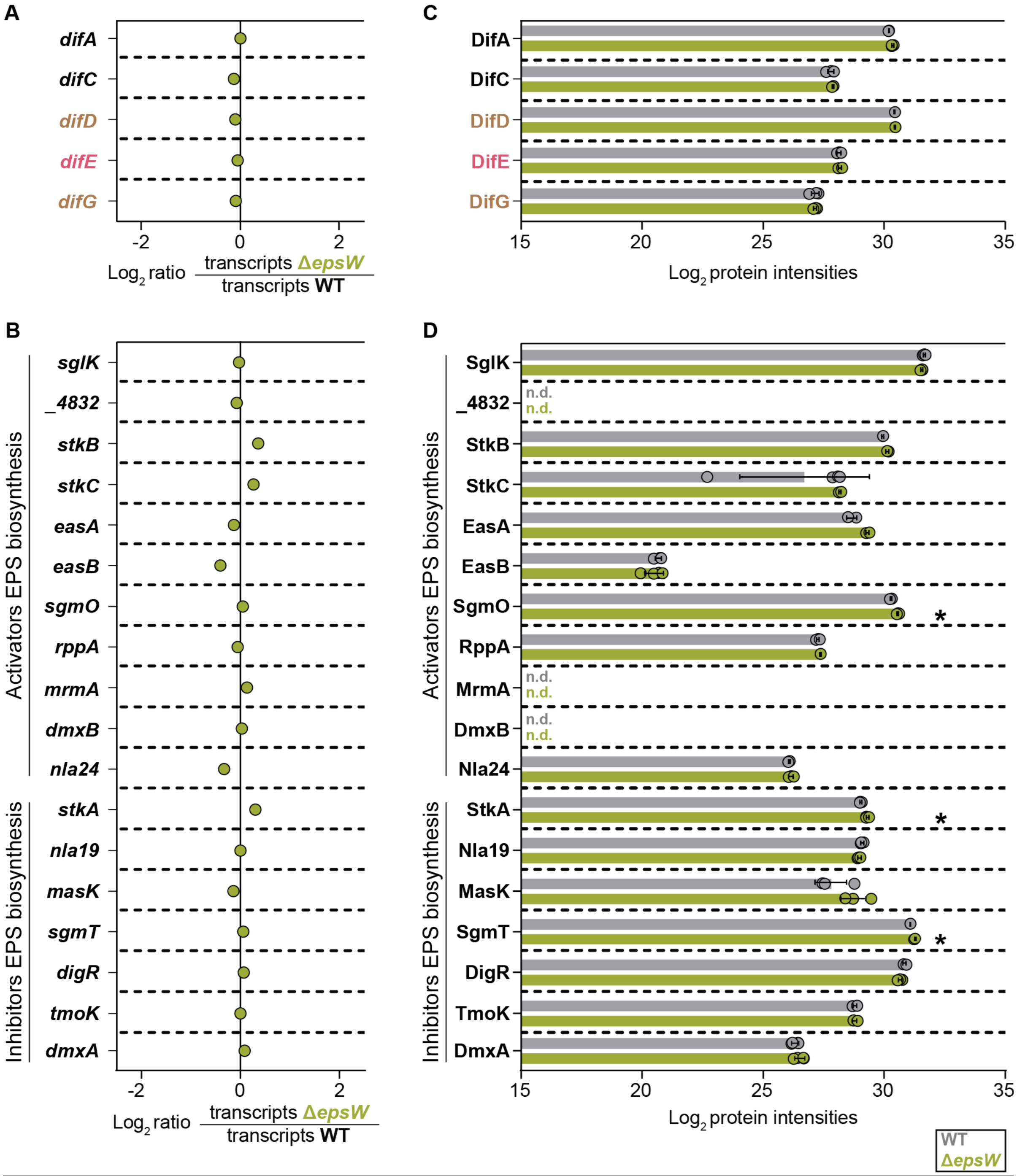

Figure S1. EpsW is neither required for the accumulation of Dif proteins nor of other regulators of EPS biosynthesis.

(A, B) Differential expression of *dif* genes (A) and other genes encoding regulators of EPS biosynthesis (B) in the Δ*epsW* mutant compared to WT. The RNAseq experiment was performed with four biological replicates per strain from cells grown in suspension culture. X-axis, log_2-fold ratio of the mean transcripts in the Δ*epsW* mutant over the mean transcripts in the WT calculated using the DESeq2 method (1). Statistical analysis was performed in the DESeq2 analysis. No significant differences were identified (adjusted *P* value ≤ 0.001). For detailed descriptions of the regulators in B see (2).

(C, D) Protein abundance in whole-cell proteomes of the Δ*epsW* mutant compared to WT. The LFQ-MS-based proteomics was performed with four biological replicates from cells grown as

in (A, B). X-axis, normalized $\log_2$ intensities of proteins in the indicated strains. Data points represent each of the four biological replicates. Error bars, mean ± standard deviation (SD) based on these replicates. *, $P < 0.001$ (Welch's test); n. d., not detected.

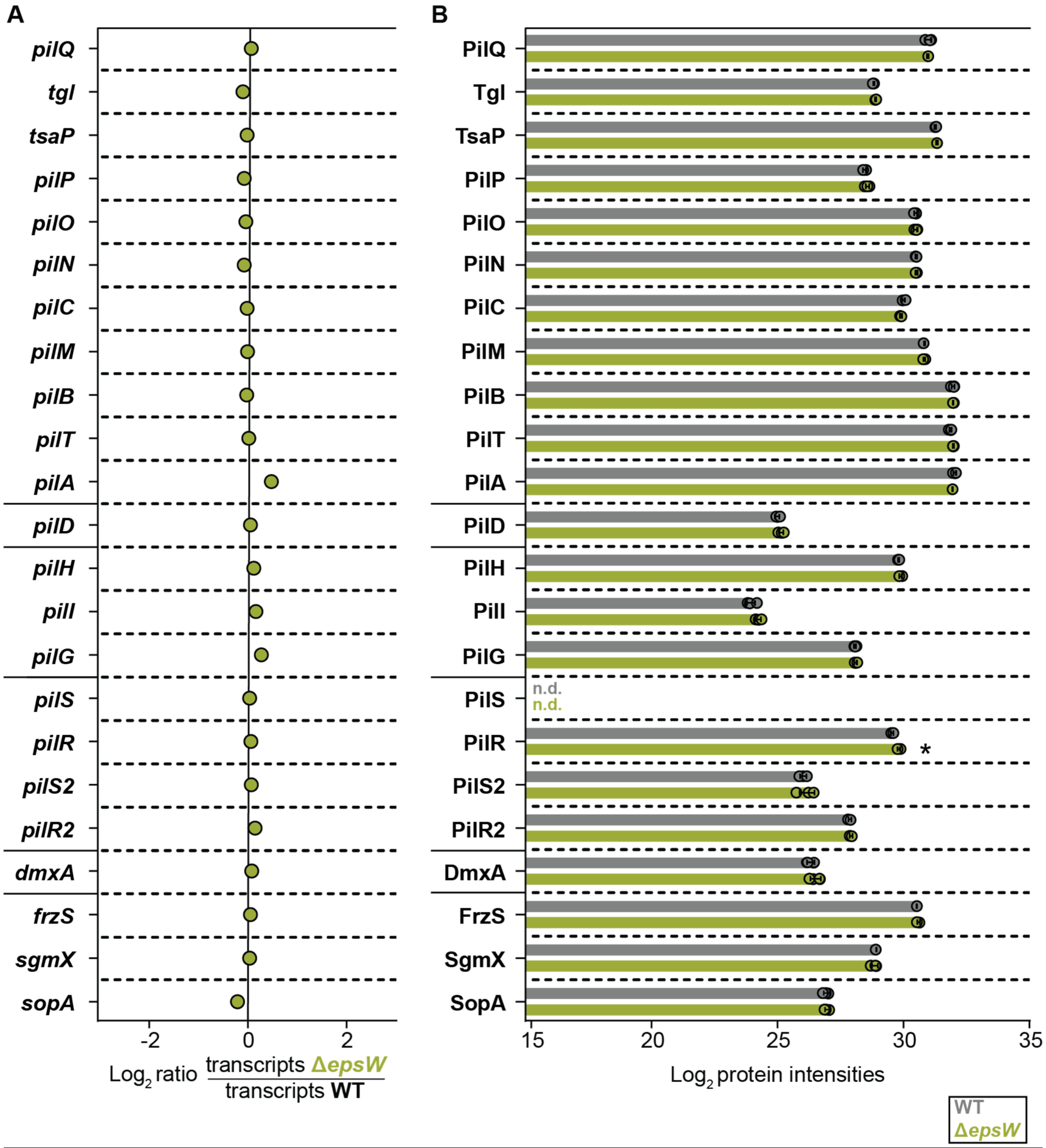

Figure S2. EpsW is not required for the accumulation of proteins required for T4P formation and function.

(A) Differential expression of genes encoding proteins required for T4P formation and function in the Δ*epsW* mutant compared to WT. The RNAseq experiment was performed with four biological replicates per strain from cells grown in suspension culture. X-axis, log_2-fold ratio of the mean transcripts in the Δ*epsW* mutant over the mean transcripts in the WT calculated using the DESeq2 method (1). Statistical analysis was performed in the DESeq2 analysis. No significant differences were identified (adjusted *P* value ≤ 0.001). PilQ is the multimeric OM secretin stabilized by LysM-domain protein TsaP (3-6). Tgl stimulates PilQ multimerization (5, 6). PilN/-O/-P are structural components in the periplasm. PilC/-M form the IM/cytoplasmic platform complex. PilB/-T are the extension and retraction ATPases, respectively, and PilA is the major pilin (4, 7). PilH/-I/-G are suggested to form an ABC transporter (8). PilD is the prepilin leader peptidase (8, 9). PilR/-S/-R2/-S2 are regulatory proteins (10, 11). DmxA is the diguanylate cyclase important for stimulating c-di-GMP synthesis during cytokinesis (12). FrzS, SgmX, and SopA jointly regulate T4P formation (13-16).

(B) Protein abundance in whole-cell proteomes of the Δ*epsW* mutant compared to WT. The LFQ-MS-based proteomics was performed with four biological replicates from cells grown as

in (A). X-axis, normalized log_2 intensities of proteins in the indicated strains. Data points represent each of the four biological replicates. Error bars, mean ± SD. *, $P < 0.001$ (Welch’s test); n.d., not detected.

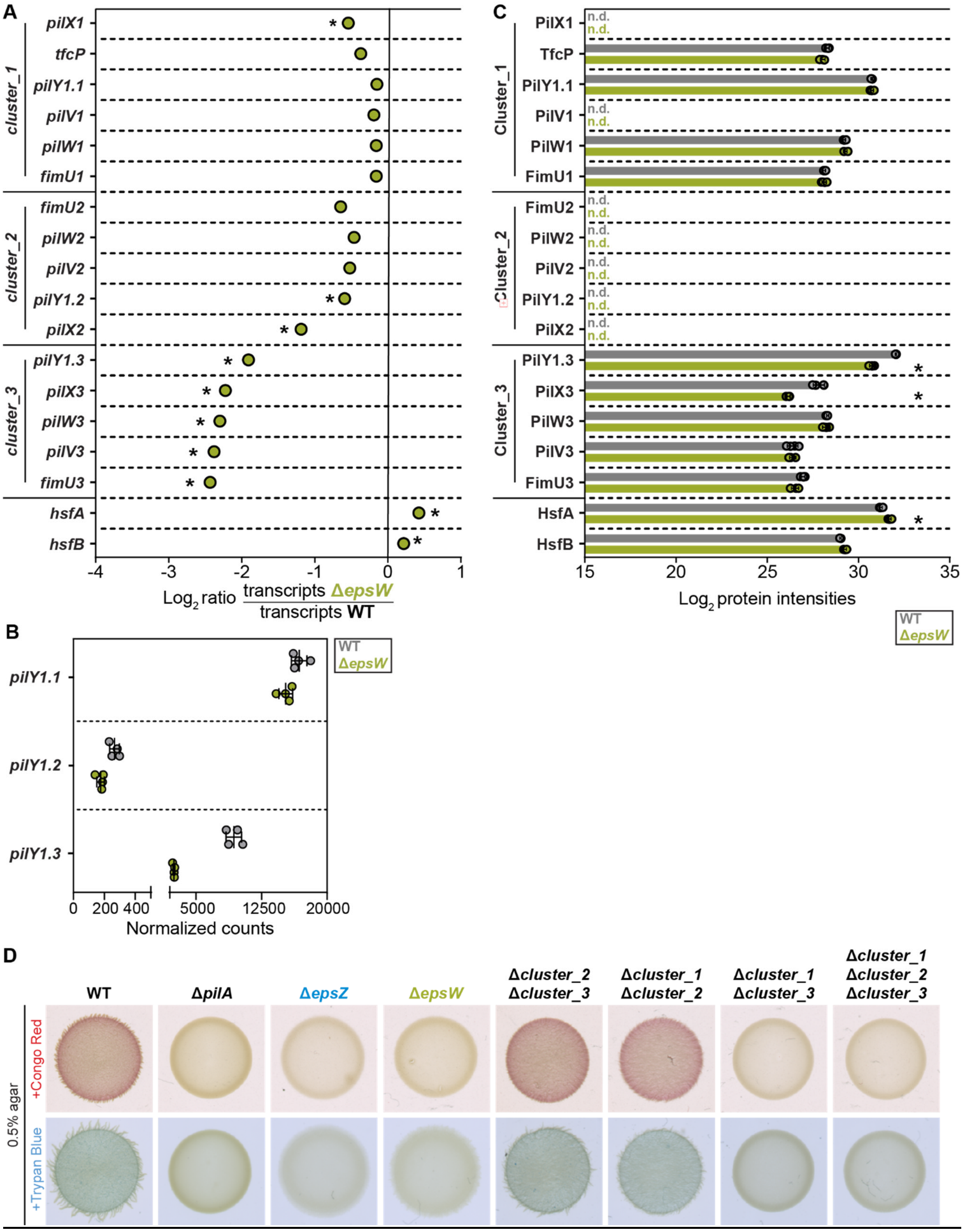

<u>Figure S3. EpsW is important for the accumulation of PilY1.3 and PilX3.</u>

(A) Differential expression of genes encoding cluster_1, cluster_2 and cluster_3 T4P priming complexes in the Δ*epsW* mutant compared to the WT. The RNAseq experiment was performed with four biological replicates per strain from cells grown in suspension culture. X-axis, $\log_2$-fold ratio of the mean transcripts in the Δ*epsW* mutant over the mean transcripts

in the WT calculated using the DESeq2 method (1). Statistical analysis was performed in the DESeq2 analysis. *, adjusted *P*-value ≤0.001.
(B) Normalized read counts for *pilY1.1*, *pilY1.2* and *pilY1.3* in the RNAseq experiment of the Δ*epsW* mutant and the WT.
(C) Protein abundance in whole-cell proteomes of the Δ*epsW* mutant compared to WT. The LFQ-MS-based proteomics was performed with four biological replicates from cells grown as in (A). X-axis, normalized $\log_2$ intensities of the indicated proteins in the indicated strains. Data points represent each of the four biological replicates. Error bars, mean ± SD. *, $P < 0.001$ (Welch's test); n.d., not detected.
(D) Cluster_1 encoding minor pilins and PilY1.1 is sufficient for EPS biosynthesis. EPS biosynthesis was assessed by spotting cells on 0.5% agar supplemented with 0.5% CTT and either Congo red or Trypan blue, and images were recorded after 24 h. The Δ*epsZ* mutant was used as the negative control for EPS biosynthesis. For comparison, the Δ*pilA* mutant, which lacks the major pilin of T4P (11), was included. Similarly, different cluster mutants were included, demonstrating that cluster_3 is also sufficient for EPS biosynthesis, while cluster_2 is not.

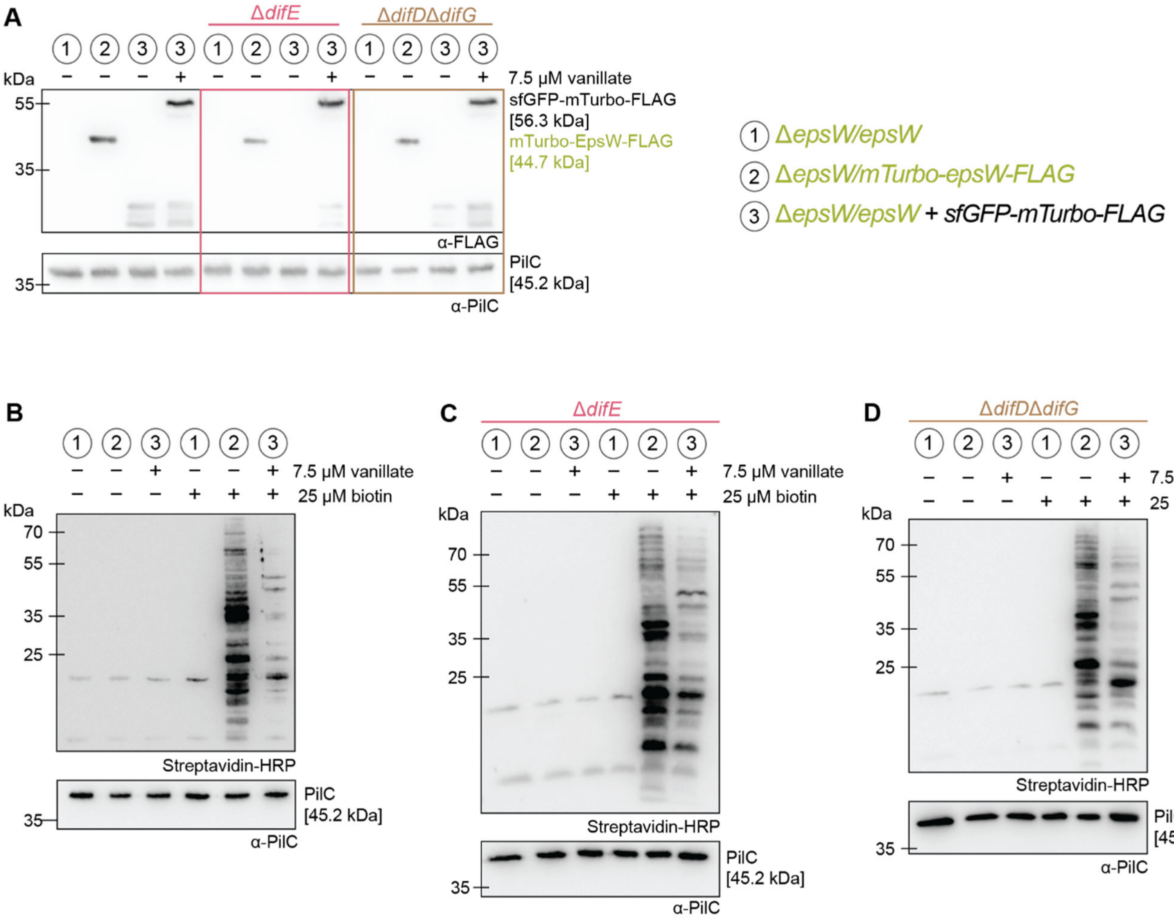

<u>Figure S4. mTurbo-EpsW-FLAG and sfGFP-mTurbo-Flag accumulate and have biotin ligase activity *in vivo*</u>

(A) Immunoblots of mTurbo-EpsW-FLAG and sfGFP-mTurbo-FLAG abundance. Total cell lysates from an equal amount of cells of the indicated strains were separated by SDS-PAGE, followed by immunoblotting with α-FLAG antibodies. The upper blot was stripped and reprobed with α-PilC antibodies as a loading control. +/− symbols indicate whether 7.5 μM vanillate was added or not for 18 h. The code for the different strains is indicated on the right. *epsW* and *mTurbo-epsW-FLAG* were ectopically expressed from the *pilA* promoter from a plasmid integrated in a single copy at the Mx8 *attB* site. *sfGFP-mTurbo-FLAG* was expressed from the vanillate-inducible promoter from a plasmid integrated in a single copy at the *18-19* locus.

(B-D) The mTurbo constructs have biotin ligase activity. Total cell lysates from an equal amount of cells of the indicated strains were analyzed by SDS-PAGE, followed by blotting and detection using Streptavidin-HRP. The blots were subsequently stripped and reprobed with α-PilC antibodies as a loading control. +/− symbols indicate whether 7.5 μM vanillate was added or not for 18 h and whether they were incubated with 25 μM biotin for 3 h. The code for the different strains is as in A. Note that the mTurbo constructs had low biotin-ligase activity when grown without biotin, indicating low endogenous biotinylation of proteins.

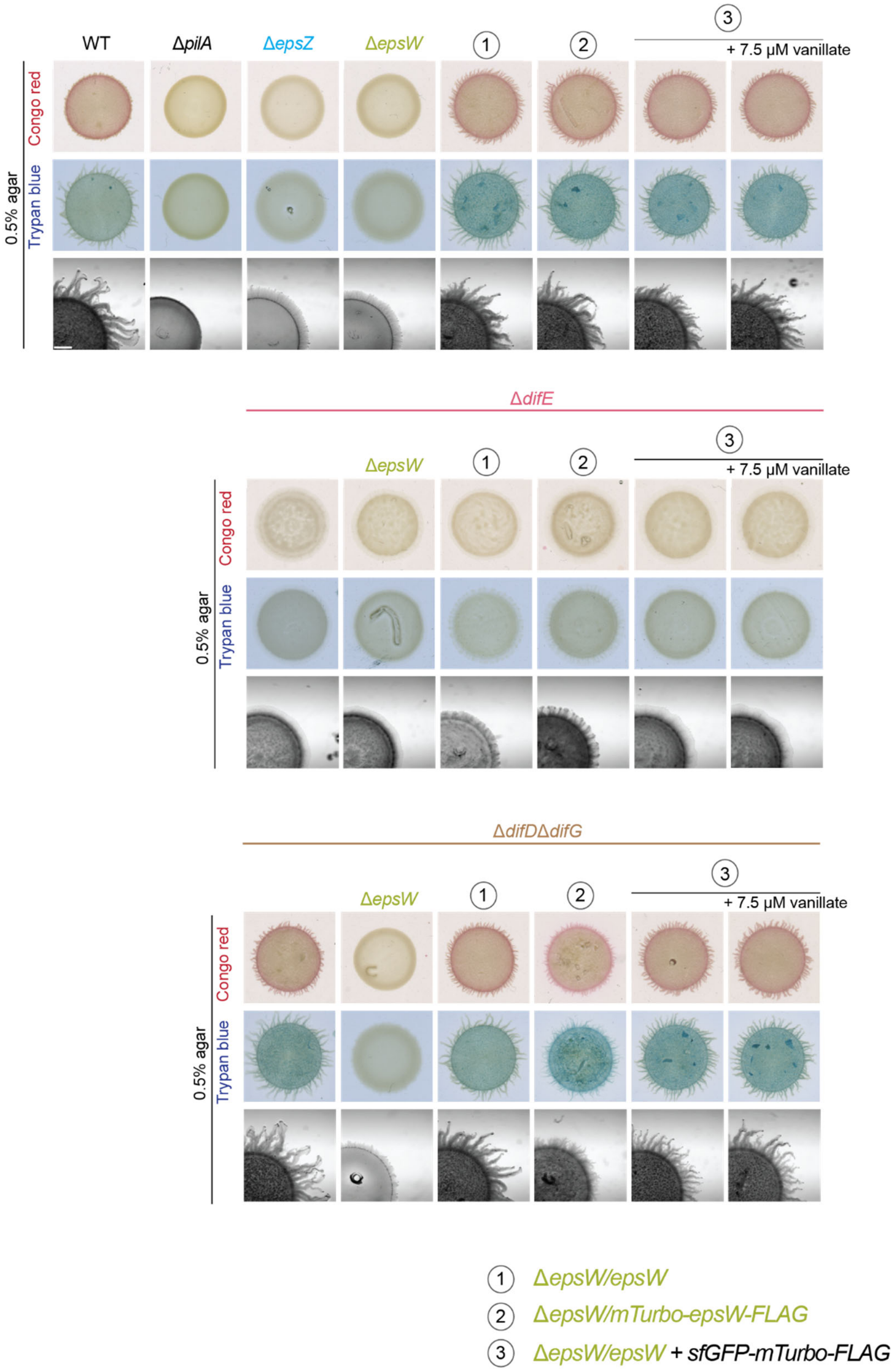

Figure S5. mTurbo-EpsW-FLAG is functional

EPS biosynthesis and T4P-dependent motility in the indicated strains. EPS biosynthesis was assessed on 0.5% agar containing 0.5% CTT and either Congo red or Trypan blue, and images were captured after 24 h. As the negative control for EPS biosynthesis, the Δ*epsZ* mutant was used. T4P-dependent motility was analyzed on 0.5% agar with 0.5% CTT, with images recorded after 24 h. The Δ*pilA* mutant, which lacks the major subunit of T4P (11), was

used as the negative control for T4P-dependent motility. The code for the different strains is indicated below. Scale bar, 1 mm.

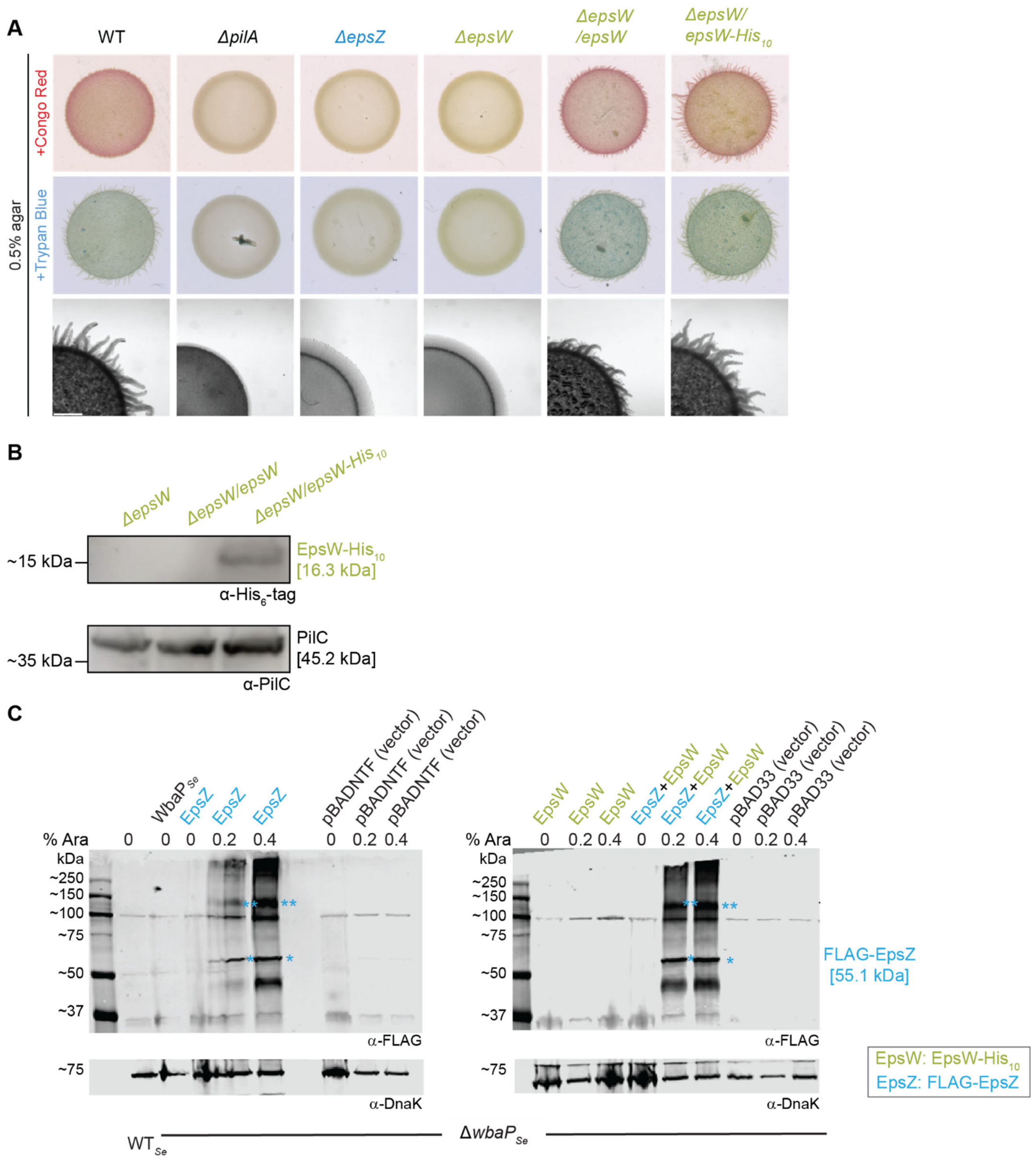

Figure S6. EpsW-His$_{10}$ is functional and accumulates in *M. xanthus* and FLAG-EpsZ accumulates in *S. enterica*.

(A) EPS biosynthesis and T4P-dependent motility in the indicated *M. xanthus* strains. EPS biosynthesis was assessed on 0.5% agar containing 0.5% CTT and either Congo red or Trypan blue, and images were captured after 24 h. As the negative control for EPS biosynthesis, the Δ*epsZ* mutant was used. T4P-dependent motility was analyzed on 0.5% agar with 0.5% CTT, with images recorded after 24 h. The Δ*pilA* mutant, which lacks the major subunit of T4P (11), was used as the negative control for T4P-dependent motility. In the

Δ*epsW/epsW* strain and the Δ*epsW/epsW-His*$_{10}$ strains, the respective genes were ectopically expressed from the *pilA* promoter from plasmids integrated in a single copy at the Mx8 *attB* site. Scale bar, 1 mm.
(B) Immunoblot analysis of EpsW-His$_{10}$. Total cellular lysates from an equal amount of cells of the indicated *M. xanthus* strains were separated *via* SDS-PAGE, followed by immunoblotting with α-His$_6$-tag antibodies. The upper blot was stripped and reprobed with α-PilC antibodies as loading control.
(C) Immunoblot analysis of FLAG-EpsZ abundance in the *S. enterica* Δ*wbaP* mutant (Δ*wbaP$_{Se}$*). The indicated proteins were expressed as in the experiment to detect LPS O-antigen in the presence of arabinose as indicated. Total cellular lysates from an equal amount of cells of the indicated *S. enterica* strains were separated by SDS-PAGE and probed with α-FLAG antibodies and α-DnaK antibodies as a loading control. * and ** indicate the monomeric and oligomeric forms of FLAG-EpsZ. Upper and lower panels are from the blots. All lysates were prepared in the same experiment and separated on by SDS-PAGE on two gels. Note that WbaP of *S. enterica* (WbaP$_{Se}$) is not FLAG-tagged and, therefore, not detected. FLAG-EpsZ and EpsW-His$_{10}$ were expressed from pMP146 (vector: pBADNTF) and pJSc143 (vector pBAD33), respectively under the control of an arabinose-inducible promoter.

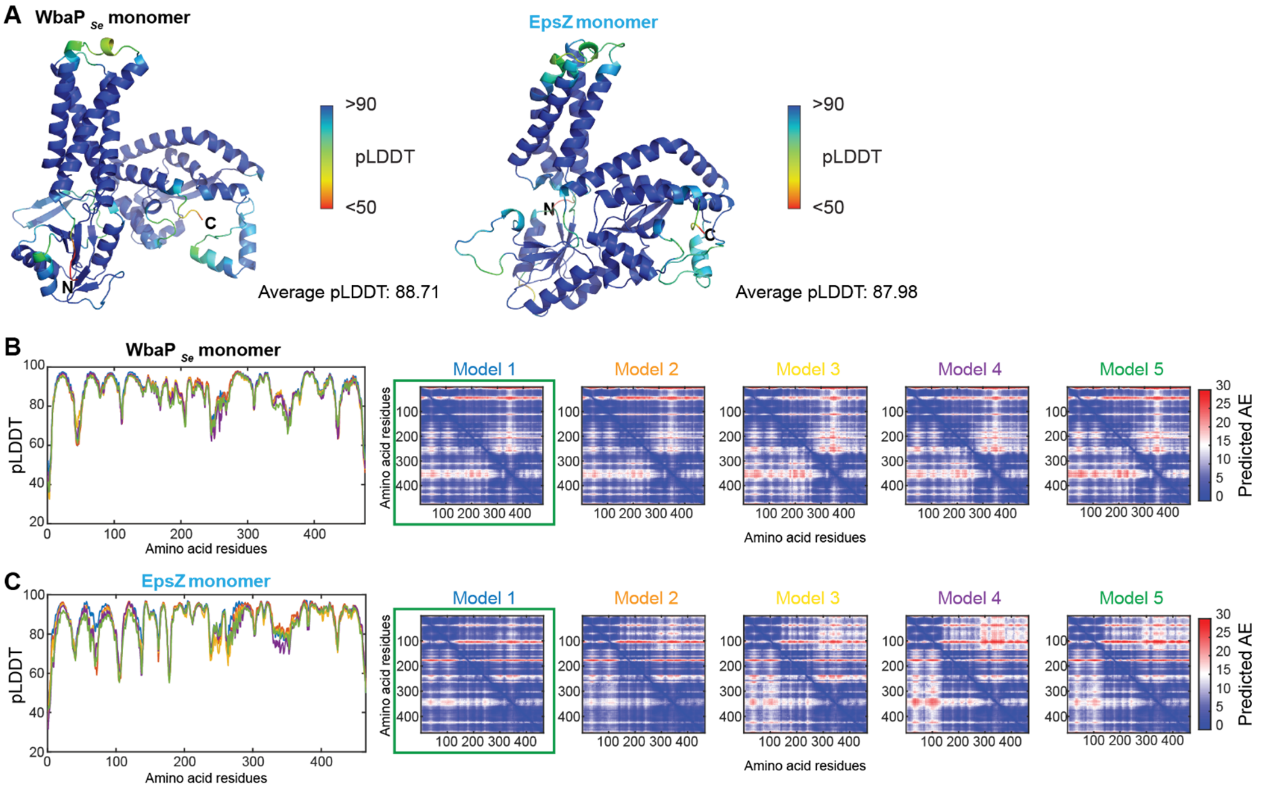

Figure S7. AlphaFold2 models of monomeric WbaP$_{Se}$ and EpsZ.

(A) Left panel, model rank 1 of WbaP$_{Se}$ colored according to pLDDT score, with the average pLDDT indicated. Right panel, model rank 1 of EpsZ colored according to pLDDT score, with the average pLDDT indicated. N- and C-termini are shown.

(B) Left panel, pLDDT plot shown for the five generated models of WbaP$_{Se}$. Right panel, pAE plots shown for the five generated models of WbaP$_{Se}$. Model rank 1 (highlighted by a green box) was selected for further analyses.

(C) Left panel, pLDDT plot shown for the five generated models of EpsZ. Right panel, pAE plots shown for the five generated models of EpsZ. Model rank 1 (highlighted by a green box) was selected for further analyses.

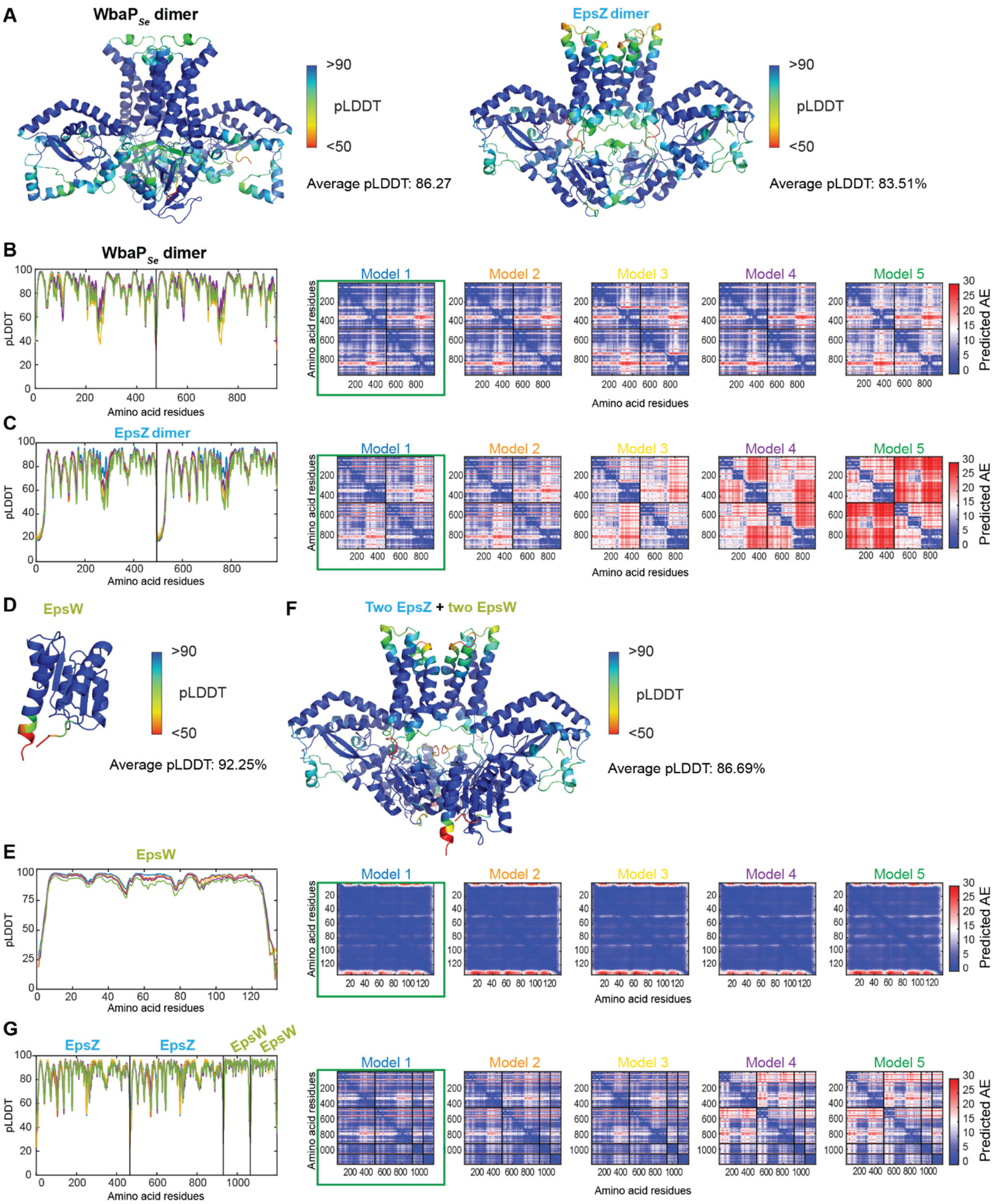

Figure S8. AlphaFold2-Multimer models of the WbaP$_{Se}$ dimer, the EpsZ dimer, and the EpsZ-EpsW heterocomplex in 2:2 stoichiometry.

(A) Left panel, model rank 1 of the WbaP$_{Se}$ dimer colored according to pLDDT score, with average pLDDT indicated. Right panel, model rank 1 of the EpsZ dimer colored according to pLDDT score, with average pLDDT indicated.

(B) Left panel, pLDDT plot shown for the five generated models of the WbaP$_{Se}$ dimer. Right panel, pAE plots shown for the five generated models of the WbaP$_{Se}$ dimer. Model rank 1 (highlighted by a green box) was selected for further analyses.
(C) Left panel, pLDDT plot shown for the five generated models of the EpsZ dimer. Right panel, pAE plots shown for the five generated models of the EpsZ dimer. Model rank 1 (highlighted by a green box) was selected for further analyses.
(D) Model rank 1 of EpsW colored according to pLDDT score, with average pLDDT indicated.
(E) Left panel, pLDDT plot shown for the five generated models of EpsW. Right panel, pAE plots shown for the five generated models of EpsW. Model rank 1 (highlighted by a green box) was selected for further analyses.
(F) Model rank 1 of the EpsZ-EpsW heterocomplex in 2:2 stoichiometry colored according to pLDDT score, with average pLDDT indicated.
(G) Left panel, pLDDT plot shown for the five generated models of the EpsZ-EpsW heterocomplex in 2:2 stoichiometry. Right panel, pAE plots shown for the five generated models of the EpsZ-EpsW heterocomplex in 2:2 stoichiometry. Model rank 1 (highlighted by a green box) was selected for further analyses.

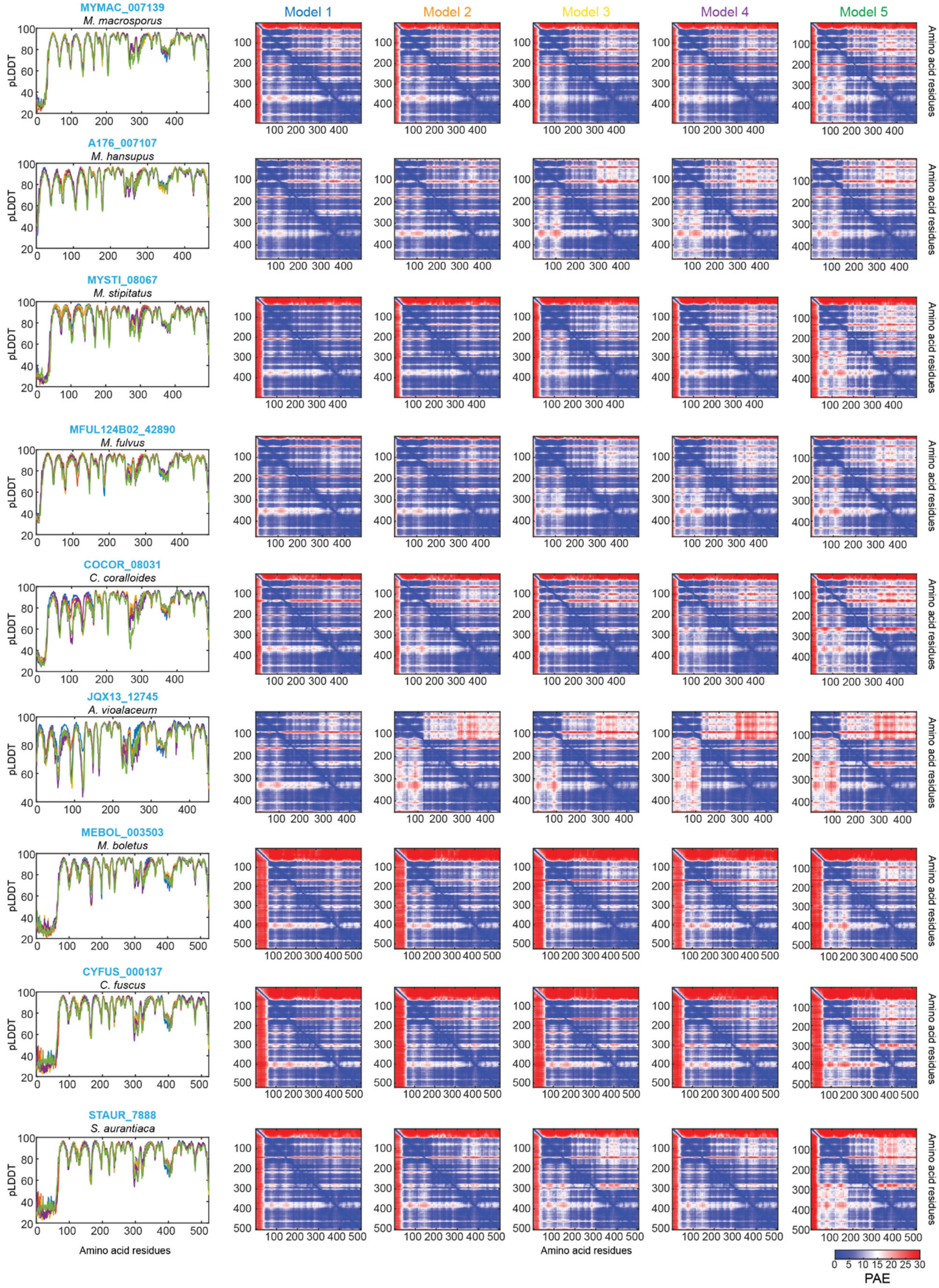

Figure S9. AlphaFold2 models of monomers of nine myxobacterial EpsZ orthologs encoded together with an EpsW ortholog.

Left panels, pLDDT plot shown for the five generated models of each EpsZ ortholog. Right panels, pAE plots shown for the five generated models of each EpsZ ortholog. Model rank 1 were selected for further analyses. Of note, in some models, the N-terminus is unstructured and has low confidence.

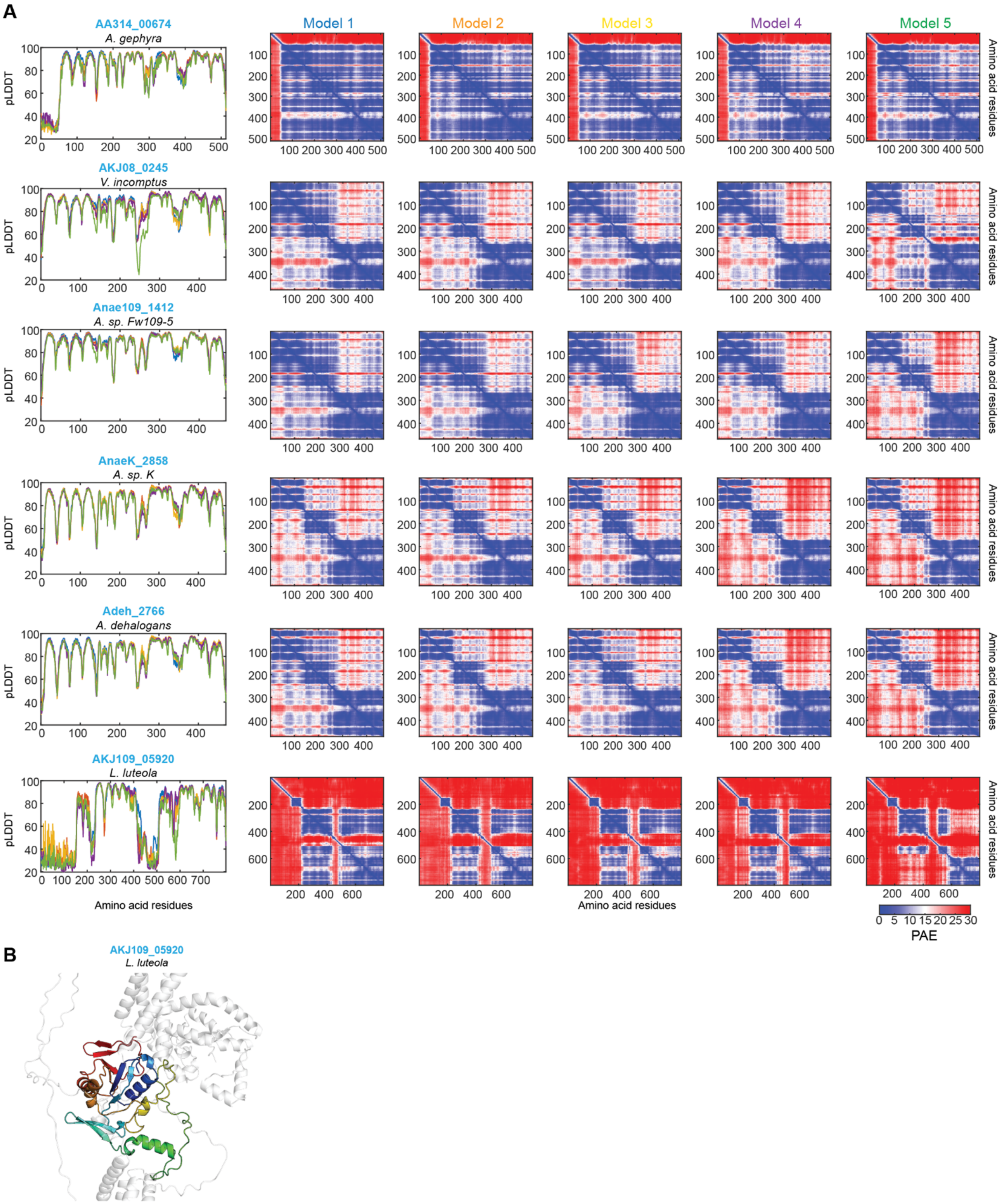

Figure 10. AlphaFold2 models of six myxobacterial EpsZ orthologs encoded without an EpsW ortholog.

(A) Left panels, pLDDT plot shown for the five generated models of each EpsZ ortholog. Right panels, pAE plots shown for the five generated models of each EpsZ ortholog. Model rank 1 were selected for further analyses. Of note, in some models, the N-terminus is unstructured and has low confidence.

(B) Computational structural model of the EpsZ ortholog AKJ109_05920 in *Labilithrix luteola*. The cytoplasmic DUF of the rank 1 AlphaFold2 model is shown and depicted using a gradient from blue (N-terminus) to red (C-terminus).

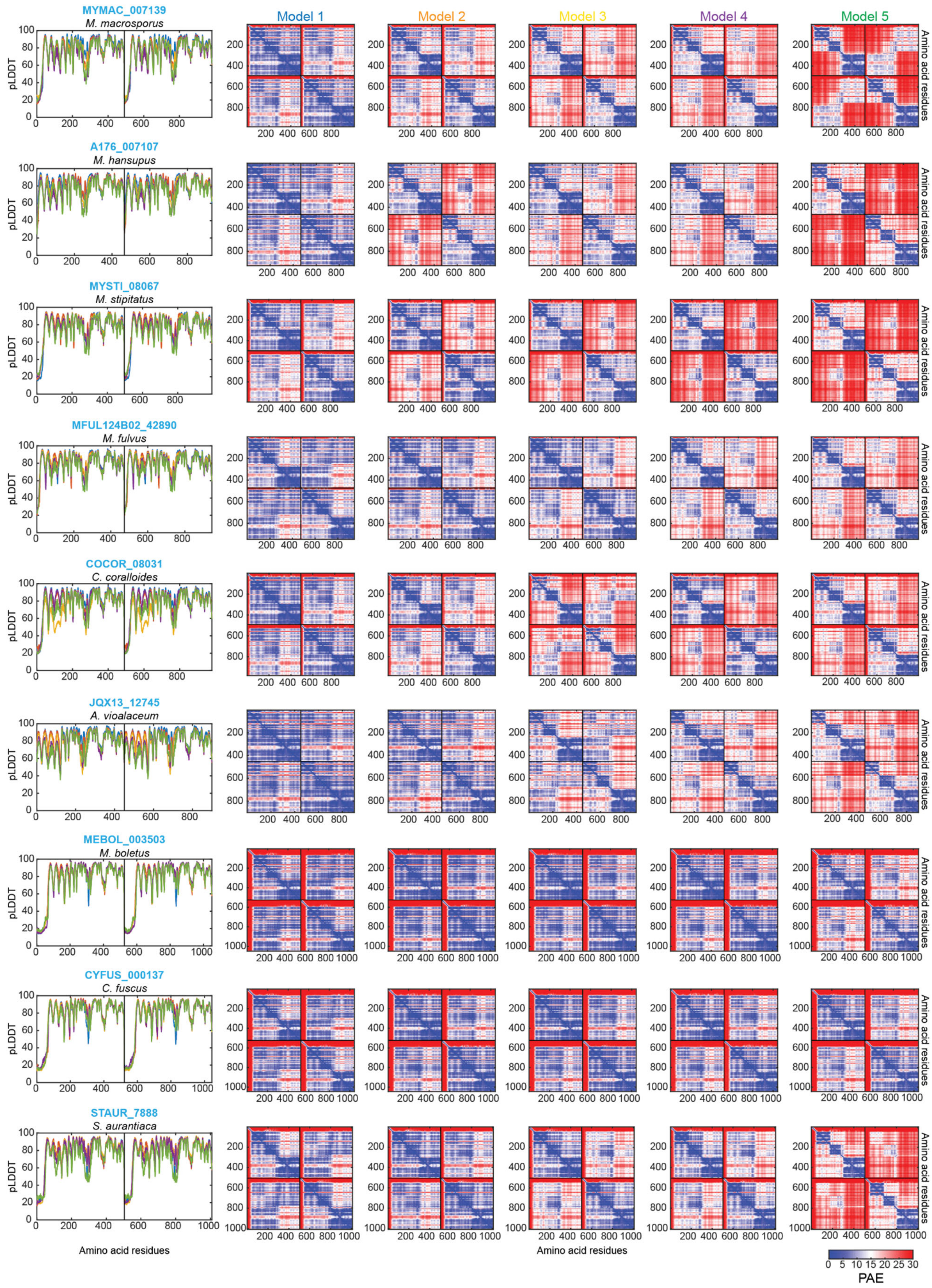

Figure S11. AlphaFold2-Multimer models of dimers of nine myxobacterial EpsZ orthologs encoded together with an EpsW ortholog.

Left panels, pLDDT plot shown for the five generated dimeric models of each EpsZ ortholog. Right panels, pAE plots shown for the five generated dimeric models of each EpsZ ortholog. Model rank 1 were selected for further analyses.

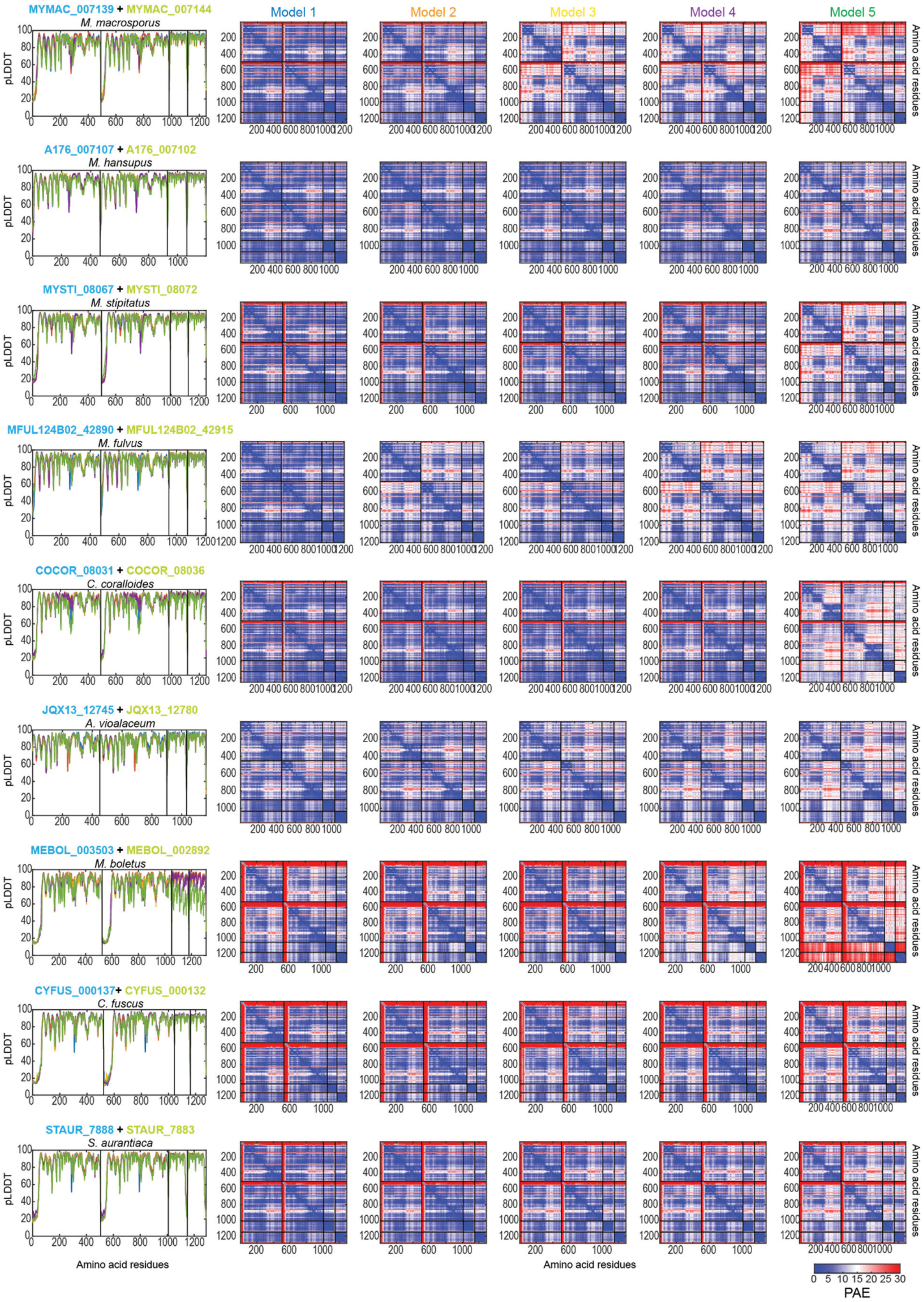

Figure S12. AlphaFold2-Multimer models of dimers of heterocomplexes of nine myxobacterial EpsZ and EpsW orthologs in 2:2 stoichiometry.

Left panels, pLDDT plot shown for the five generated models of each heterocomplex (2:2 stoichiometry) composed of myxobacterial orthologs of EpsZ and EpsW. Right panels, pAE plots shown for these models. Model rank 1 were selected for further analyses.

Table S1 Genes with significantly changed transcript levels in the Δ*epsW* mutant compared to WT.

Gene	Function	Log_2 ratio[1]	−Log_{10} *P*-value
MXAN_0504	Hypothetical protein	-2.64	155.0
MXAN_1370	Hypothetical protein	-2.64	88.2
fimU3	Minor pilin	-2.38	71.1
pilV3	Minor pilin	-2.37	39.0
pilW3	Minor pilin	-2.29	180.1
pilX3	Minor pilin	-2.22	56.9
MXAN_7017	Csd3, CRISPR-associated	2.03	3.1
MXAN_7020	Cas3, CRISPR-associated	2.13	4.1
MXAN_7018	Csd1, CRISPR-associated	2.39	3.4
MXAN_7019	Cas51, CRISPR-associated	2.99	4.2

[1] Numbers indicate Log_2 ratio of the mean transcripts in the Δ*epsW* mutant over the mean transcripts in the WT calculated using the DESeq2 method based on four biological replicates per strain. Criteria for significant changes: Log_2 (FC) ≥ 2.00 or ≤ -2.00 and -Log_{10} (*P*-value) ≥ 3.0. Note that in this global analysis, we used stringent fold-change cut-offs for quantitative analysis, while in Figure 1C, Figure S1A and B, Figure S2A, and Figure S3A, no fold-change cut-off was used.

Table S2 Proteins with significantly changed accumulation levels in the Δ*epsW* mutant compared to WT.

Protein	Function	Log_2 ratio[1]	$-Log_{10}$ *P*-value
FibA	Matrix-associated zinc metalloprotease FibA	-5.89	8.6
MXAN_4534	Chitinase, class I	-4.14	7.1
MsrBA	Methionine-R-sulfoxide reductase/methionine-S-sulfoxide reductase	-3.95	3.5
MXAN_1651	Uncharacterized protein	-3.86	6.6
MXAN_3676	Uncharacterized protein	-3.85	9.0
MXAN_6837	Putative lipoprotein	-3.77	4.4
MXAN_0793	Putative lipoprotein	-3.70	8.7
MXAN_2837	Putative lipoprotein	-3.56	4.9
MXAN_7464	Uncharacterized protein	-3.17	4.3
MXAN_1967	Putative peptidase. S8 (Subtilisin) family	-2.99	5.8
MXAN_0225	Putative long-chain-fatty-acid-CoA ligase	-2.80	4.0
MXAN_1672	Uncharacterized protein	-2.76	5.0
MXAN_1287	Conserved domain protein	-2.74	4.4
MXAN_4411	JmjC domain protein	-2.65	3.8
MXAN_0201	Hydrolase. alpha/beta fold family	-2.63	3.4
MXAN_2903	Putative lipoprotein	-2.59	3.8
MXAN_6709	Putative lipoprotein	-2.35	3.8
MXAN_7070	Uncharacterized protein	-2.34	3.5
MXAN_4416	Cephalosporin hydroxylase family protein	-2.21	3.3
MXAN_4524	Uncharacterized protein	-2.15	5.1
MXAN_5308	Putative lipoprotein	-2.14	5.1
MXAN_6116	Uncharacterized protein	-2.04	3.8
MXAN_7039	Putative lipoprotein	2.32	8.8
MXAN_3114	DnaJ domain protein	2.43	4.4
MXAN_5560	Cytochrome c family protein	2.47	3.7
MXAN_2579	Metallophosphoesterase/PKD domain protein	2.83	3.5
MXAN_5456	Uncharacterized protein	3.70	4.5

[1] Log_2 ratio of the mean protein intensities in the Δ*epsW* mutant over the mean protein intensities in the WT calculated based on four biological replicates per strain. Criteria for significant changes: Log_2 (FC) ≥ 2.00 or ≤ -2.00 and $-Log_{10}$ (*P*-value) ≥ 3.0. Note that in this global analysis, we used stringent fold-change cut-offs for quantitative analysis, while in Figure 1C, Figure S1A and B, Figure S2A, and Figure S3A, no fold-change cut-off was used.

Table S3. Surface accessible, cytoplasmic Lys residues in Eps proteins and DifE[1].

Protein	Function	Predicted subcellular localization	Surface-accessible, cytoplasmic Lys residues
EpsZ	Large monoPGT	IM	13
Wzx_{EPS}	Wzx-like flippase	IM	3
EpsY	^{D1D2}OPX	Periplasm	0
EpsX	Integral OM 18-stranded β-barrel protein	OM	0
EpsW	Single-domain response regulator	Cytoplasm	5
EpsV	PCP	IM	2
EpsU	GT	IM	12
EpsH	GT	Cytoplasm	18
Wzy_{EPS}	Wzy-like polymerase	IM	2
EpsE	GT	Cytoplasm	13
EcpK	BY pseudokinase	Cytoplasm	10
EpsD	GT	Cytoplasm	7
EpsA	GT	Cytoplasm	11
DifE	Histidine protein kinase	Cytoplasm	17

Table S4. Proteins significantly enriched in miniTurbo-EpsW-FLAG proximity labeling experiments in the otherwise WT strain[1].

Locus Tag	Name	Annotation	Log_2 ratio[2]	$-Log_{10}$ *P*-value
MXAN_7420	EpsW	Single-domain response regulator	11.2	6.3
MXAN_0631		Transcriptional regulator, AraC family	7.1	5.6
MXAN_2994		Glycosyl hydrolase, family 57	5.0	6.0
MXAN_3056		Uncharacterized protein	4.5	4.7
MXAN_6692	DifE	Histidine protein kinase, Dif system	4.2	4.5
MXAN_6931		Thioredoxin	4.2	4.8
MXAN_7415	EpsZ	Phosphoglycosyl transferase	4.0	4.4
MXAN_4666		General secretion pathway protein E, N-terminal domain protein	3.7	6.2
MXAN_5807		Putative membrane protein	3.7	4.0
MXAN_4576		Acetyltransferase, GNAT family	3.6	5.6
MXAN_2578		Methyltransferase, RsmB/NOP family	3.1	4.7

[1] EpsW is indicated in green, DifE and EpsZ together with the two other proteins that were enriched in the otherwise WT and the Δ*difD*Δ*difG* strains but not in the Δ*difE* strain are marked in orange and yellow, respectively. Proteins potentially involved in monosaccharide synthesis or modification are marked in blue.

[2] Log_2-fold ratios of mean protein intensities in mTurbo-EpsW-FLAG samples relative to the sfGFP-mTurbo-FLAG samples based on four biological replicates. Significantly enriched proteins fulfill the criteria $\log_2$ fold ratio ≥3.0; $-\log_{10}$ *P*-value ≥4.0.

Table S5. Proteins significantly enriched in miniTurbo-EpsW-FLAG proximity labeling experiments in the Δ*difE* background[1].

**Locus Tag**	**Name**	**Annotation**	**Log_2 ratio**[2]	**$-Log_{10}$ *P*-value**
MXAN_4082	FusA 3	Elongation factor G3	13.2	8.8
MXAN_7420	EpsW	Single-domain response regulator	12.2	4.8
MXAN_3079		Phasin family protein	5.4	4.8
MXAN_2993		Conserved domain protein	5.3	4.7
MXAN_6798		Type I restriction enzyme R protein N-terminal domain-containing protein	4.6	6.0
MXAN_4666		General secretion pathway protein E. N-terminal domain protein	4.2	6.5
MXAN_3986		ABC transporter, ATP-binding protein	3.6	5.5
MXAN_0731		Tryptophan 2.3-dioxygenase	3.4	4.4
MXAN_6931		Thioredoxin	3.2	4.1
MXAN_3943		Cytochrome P450 family protein	3.1	5.2
MXAN_6765		ABC transporter, ATP-binding protein	3.0	8.0

[1] EpsW is indicated in green.

[2] Log_2-fold ratios of mean protein intensities in mTurbo-EpsW-FLAG samples relative to the sfGFP-mTurbo-FLAG samples based on four biological replicates. Significantly enriched proteins fulfill the criteria log_2 fold ratio ≥3.0; $-log_{10}$ *P*-value ≥4.0,

Table S6. Proteins significantly enriched in miniTurbo-EpsW-FLAG proximity labeling experiments in the Δ*difD*Δ*difG* background[1].

Locus Tag	Name	Annotation	Log_2 ratio[2]	$-Log_{10}$ *p*-value
MXAN_7420	EpsW	Single-domain response regulator	14.2	7.6
MXAN_4402		Non-ribosomal peptide synthetase	8.1	6.0
MXAN_2365		Uncharacterized protein	7.9	4.3
MXAN_7298		Cytochrome P450 family protein	7.0	4.9
MXAN_0631		Transcriptional regulator, AraC family	7.0	7.1
MXAN_3943		Cytochrome P450 family protein	6.8	4.4
MXAN_7415	EpsZ	Phosphoglycosyl transferase	5.4	4.5
MXAN_4292		Polyketide synthase	5.4	4.8
MXAN_2949		Cation ABC transporter	5.2	5.0
MXAN_2367		Acetyltransferase, GNAT family	5.0	5.6
MXAN_6883		Dienelactone hydrolase family protein	4.9	4.7
MXAN_3241		Uncharacterized protein	4.8	5.3
MXAN_5074	SpoVG	Putative septation protein SpoVG	4.4	4.1
MXAN_2977		Uncharacterized protein	4.4	5.0
MXAN_0282		Uncharacterized protein	4.3	4.4
MXAN_4730		Lipoprotein releasing system. transmembrane protein, LolC/E family	4.3	4.8
MXAN_2283	DusA	tRNA-dihydrouridine synthase	4.2	5.7
MXAN_6758		Uncharacterized protein	4.0	4.1
MXAN_4410		Cephalosporin hydroxylase family protein	3.9	4.0
MXAN_1006		Uncharacterized protein	3.9	4.2
MXAN_7398		Histidine kinase	3.8	4.3
MXAN_2788		Rieske 2Fe-2S domain protein	3.6	6.0
MXAN_2926		Ferredoxin. 2Fe-2S	3.5	4.1
MXAN_5807		Putative membrane protein	3.5	4.1
MXAN_6692	DifE	Histidine protein kinase, Dif system	3.4	6.2
MXAN_1308		Uncharacterized protein	3.4	8.1
MXAN_4735		Putative membrane protein	3.3	5.2
MXAN_3788		Uncharacterized protein	3.3	5.5
MXAN_1292		Uncharacterized protein	3.2	4.0
MXAN_0429		Uncharacterized protein	3.1	5.1
MXAN_5156		Uncharacterized protein	3.1	5.5
MXAN_4302		FAD-binding domain protein	3.1	4.3
MXAN_1566		Uncharacterized protein	3.1	4.5

[1] EpsW is indicated in green, DifE and EpsZ together with the two other proteins that were enriched in the otherwise WT and the Δ*difD*Δ*difG* strains but not in the Δ*difE* strain are marked in orange and yellow, respectively. Proteins potentially involved in monosaccharide synthesis or modification are marked in blue.

[2] Log_2-fold ratios of mean protein intensities in mTurbo-EpsW-FLAG samples relative to the sfGFP-mTurbo-FLAG samples based on four biological replicates. Significantly enriched proteins fulfill the criteria log_2 fold ratio ≥3.0; $-log_{10}$ *P*-value ≥4.0,

Table S7. Strains used in this work

Species and strain	Genotype	Reference or source
M. xanthus		
DK1622	WT	(17)
DK10410	Δ*pilA*	(11)
SA7400	Δ*epsZ*	(18)
SA6888	Δ*cluster*_1Δ*cluster*_2	(19)
SA6892	Δ*cluster*_2Δ*cluster*_3	(19)
SA6899	Δ*cluster*_1Δ*cluster*_3	(19)
SA7609	Δ*cluster*_1Δ*cluster*_2Δ*cluster*_3	(19)
SA5649	Δ*difE*	This work
SA7415	Δ*epsW*	This work
SA8550	Δ*epsW attB*::pMP146 *(*P_{pilA} *epsW)*	This work
SA11564	Δ*difD*Δ*difG*	This work
SA11578	Δ*difD*Δ*difG*Δ*epsW*	This work
SA11639	Δ*epsW attB*::pJSc105 *(*P_{pilA} *mTurbo-epsW-FLAG)*	This work
SA11646	Δ*epsW attB*::pJSc113 *(*P_{pilA} *epsW) 18-19*::pMH97 (*P_{van} *sfGFP-mTurbo-FLAG*)	This work
SA7425	Δ*difE*Δ*epsW*	This work
SA11645	Δ*difE*Δ*epsW attB*::pJSc105 (*P_{pilA} *mTurbo-epsW-FLAG))*	This work
SA11649	Δ*difE*Δ*epsW attB*::pJSc113 (*P_{pilA} *epsW)*	This work
SA11647	Δ*difE*Δ*epsW attB*::pJSc113 (*P_{pilA} *epsW) 18-19*::pMH97 *(*P_{van} *sfGFP-mTurbo-FLAG*)	This work
SA11644	Δ*difD*Δ*difG*Δ*epsW attB*::pJSc105 *(*P_{pilA} *mTurbo-epsW-FLAG)*	This work
SA13200	Δ*difD*Δ*difG*Δ*epsW attB*::pJSc113 *(*P_{pilA} *epsW)*	This work
SA11648	Δ*difD*Δ*difG*Δ*epsW attB*::pJSc113 *(*P_{pilA} *epsW) 18-19*::pMH97 *(*P_{van} *sfGFP-mTurbo-FLAG*)	This work
SA13207	Δ*epsW attB*::pJSc146 *(*P_{pilA} *epsW-His*$_{10}$*)*	This work
Salmonella		
LT2	*S. enterica* serovar Typhimurium, WT	S. Maloy
MSS2	LT2, Δ*wbaP*::*cat*	(20)
JMF20	LT2, Δ*wbaP*	This work
E. coli		
E. coli NEB Turbo	F' *proA*$^{+}$*B*$^{+}$ *lacI*^{q} *ΔlacZM15 / fhuA2 Δ(lac-proAB) glnV galK16 galE15 R(zgb-210::Tn10)*Tet^{S} *endA1 thi-1 Δ(hsdS-mcrB)5*	New England Biolabs

Table S8. Plasmids used in this work

Plasmid	Description	Reference or source
pBJ114	*galK* Kan^R	(21)
pSW105	Km^r P_{pilA}	(7)
pSWU30	Tet^r	(11)
pBAD33	Arabinose-inducible promoter, Cam^r	(22)
pBADNTF	pBAD24 for N-terminal FLAG fusion and with arabinose-inducible promoter, Amp^r	(23)
pMP036	pBJ114, in-frame deletion construct for *epsW* Km^r	This work
pDJS102	pBJ114, in-frame deletion construct for *difE* Km^r	This work
pJSc002	pBJ114, in-frame deletion construct for *difD* Km^r	This work
pJSc003	pBJ114, in-frame deletion construct for *difG* Km^r	This work
pJSc105	pSW105, P_{pilA} *mTurbo-epsW-FLAG* Km^r	This work
pJSc113	pSWU30, P_{pilA} *epsW* Tet^r	This work
pJSc143	pBAD33, *epsW-His$_{10}$*, Cam^r	This work
pJSc146	pSW105, P_{pilA} *epsW-His*$_{10}$	This work
pMP145	pSW105, P_{pilA} *epsW* Km^r	This work
pMP146	pBADNTF, *FLAG-epsZ* Amp^r	(18)
pMH97	pMR3690, P_{Van} *sfGFP-mTurbo-FLAG* Km^r	(24)
pSM13	pUC18, *wbaP* from *S. enterica* containing a 1-bp deletion at position 583 and a 2-bp deletion at position 645, which causes a frame shift at WbaP I194 and frame restoration at Y215, Amp^r	(20)
pCP20	FLP recombinase expression, Cam^r, Amp^r	(25)

Table S9. Oligonucleotides used in this work[1]

Primer name	Sequence 5'-3'	Brief description
7420_A	ATCGGGTACCAGTCGTGGGACAGCATCCT	For Δ*epsW*
7420_B	CCCAGTGCTGAGGACCGTGTGCAGGTC	For Δ*epsW*
7420_C	ACGGTCCTCAGCACTGGGGCCAGGAGG	For Δ*epsW*
7420_D	ATCGTCTAGACAGTACTCCACCAGCTTCGC	For Δ*epsW*
7420_E	ACCTGACGCTGACGGGGACC	For Δ*epsW*
7420_F	CACCTTCTCCTTCAGCCGGT	For Δ*epsW*
7420_G	GGTGGAGGACGCCCCCTTCT	For Δ*epsW*
7420_H	TGAGGAGCGACTGCACCCGC	For Δ*epsW*
7420_P_{pilA} fw	ATCGTCTAGAATGGAGTCCGACCTGCACAC	For complementation of *epsW*
7420_P_{pilA} rev	ATCGAAGCTTTCACGGTGCCCTCCTGGCCC	For complementation of *epsW*
mTurbo-P_{pilA}-for	GCGCTCTAGAATGATCCCGCTCCTG	For mTurbo-EpsW-FLAG
mTurbo-epsW-FLAG int for	GGCGGGGGCGGGAGCGAGTCCGACCTGCAC ACGG	For mTurbo-EpsW-FLAG
mTurbo-epsW-FLAG int rev	GCTCCCGCCCCCGCCCTTCTCGGCGCTGCGCA	For mTurbo-EpsW-FLAG
EpsW-FLAG-rev	CAGTAAGCTTTCACTTGTCGTCGTCGTCCTTGTA GTCCGGTGCCCTCCTGGCCCCAG	For mTurbo-EpsW-FLAG
EpsW-His-rev-HindIII	ACTGAAGCTTTCAGTGGTGGTGGTGATGGTGATG ATGATGATGCGGTGCCCTCCTGGCCCC	For EpsW-His_{10}
6692_A	ATCGGAATTCAGGCTGGGTGGCCTGGGAGT	For Δ*difE*
6692_B	CGGCACGTCGAGGTAGCGGGACATGTC	For Δ*difE*
6692_C	CGCTACCTCGACGTGCCGAGGTTACTG	For Δ*difE*
6692_D	ATCGTCTAGACAGCAGGTCGCGGATGCTGT	For Δ*difE*
6692_E	ACGGCGCCAGGAAAGATGAC	For Δ*difE*
6692_F	TTTCAGCGCCTTCTCATCCA	For Δ*difE*
6692_G	AAGGTGACGGCGCTGAAGCC	For Δ*difE*
6692_H	CCAGCGCTGGGCCAGCGTTT	For Δ*difE*
6693_A	AAAGGTACCTGCAAGGACGTCGGCGGC	For Δ*difD*
6693_B	CTTCTTCACGTCGACGACCAGGACCCG	For Δ*difD*
6693_C	GTCGTCGACGTGAAGAAGGTCCTGGGC	For Δ*difD*
6693_D	TTTTCTAGAAGCTTCCACCAGCTCCGC	For Δ*difD*
6693_E	GCAGAAGGTGCTCAAGGC	For Δ*difD*
6693_F	AGGGAGTTGGCCAGGTCG	For Δ*difD*
6693_G	GGACATCTTTGCGTCTGG	For Δ*difD*
6693_H	GACGATGAAGTCCGAGGC	For Δ*difD*
6691_A	TTTGGTACCGGAGGCGCCCGAGGATCG	For Δ*difG*
6691_B	CAGCAGGTCCGCGTCGCTGTGCAGGGG	For Δ*difG*
6691_C	AGCGACGCGGACCTGCTGGCGCGGCTG	For Δ*difG*
6691_D	TTTTCTAGAGCATGGTGCTCACCTGCG	For Δ*difG*
6691_E	ACACGTTGGTGAAGGACC	For Δ*difG*
6691_F	ATGTCGTCGATGGACACG	For Δ*difG*
6691_G	GCAAGGTGGACCTCTCCA	For Δ*difG*

6691_H	TCCAACACCACCACGCTG	For Δ*difG*

[1] Underlined sequences indicate restriction sites.

Table S10. Fully sequenced myxobacterial genomes used for the 16S RNA tree and gene co-occurrence analysis.

Species and strain name
Anaeromyxobacter dehalogenans 2CP-C
Anaeromyxobacter sp. Fw109-5
Anaeromyxobacter sp. K
Archangium gephyra DSM 2261
Archangium violaceum Cb SDU34
Chondromyces crocatus Cm c5
Corallococcus coralloides DSM 2259
Cystobacter fuscus DSM 52655
Haliangium ochraceum DSM 14365
Labilithrix luteola DSM 27648
Melittangium boletus DSM 14713SG
Minicystis rosea DSM 24000
Myxococcus fulvus 124B02
Myxococcus macrosporus DSM 14675
Myxococcus hansupus (*Myxococcus* sp. mixupus)
Myxococcus stipitatus DSM 14675
Myxococcus xanthus DK1622
Sandaracinus amylolyticus DSM 53668
Sorangium cellulosum So ce 56
Stigmatella aurantiaca DW4/3-1
Vulgatibacter incomptus DSM 27710

Supplementary References

1. Love MI, Huber W, Anders S. 2014. Moderated estimation of fold change and dispersion for RNA-seq data with DESeq2. Genome Biol 15:550.

2. Pérez-Burgos M, Søgaard-Andersen L. 2020. Biosynthesis and function of cell-surface polysaccharides in the social bacterium *Myxococcus xanthus*. Biol Chem 401:1375-1387.

3. Siewering K, Jain S, Friedrich C, Webber-Birungi MT, Semchonok DA, Binzen I, Wagner A, Huntley S, Kahnt J, Klingl A, Boekema EJ, Søgaard-Andersen L, van der Does C. 2014. Peptidoglycan-binding protein TsaP functions in surface assembly of type IV pili. Proc Natl Acad Sci U S A 111:E953-61.

4. Chang YW, Rettberg LA, Treuner-Lange A, Iwasa J, Søgaard-Andersen L, Jensen GJ. 2016. Architecture of the type IVa pilus machine. Science 351:aad2001.

5. Herfurth M, Pérez-Burgos M, Søgaard-Andersen L. 2023. The mechanism for polar localization of the type IVa pilus machine in *Myxococcus xanthus*. mBio 14:e0159323.

6. Wall D, Kolenbrander PE, Kaiser D. 1999. The *Myxococcus xanthus pilQ* (*sglA*) gene encodes a secretin homolog required for type IV pilus biogenesis, social motility, and development. J Bacteriol 181:24-33.

7. Jakovljevic V, Leonardy S, Hoppert M, Søgaard-Andersen L. 2008. PilB and PilT are ATPases acting antagonistically in type IV pilus function in *Myxococcus xanthus*. J Bacteriol 190:2411-21.

8. Wu SS, Wu J, Cheng YL, Kaiser D. 1998. The *pilH* gene encodes an ABC transporter homologue required for type IV pilus biogenesis and social gliding motility in *Myxococcus xanthus*. Mol Microbiol 29:1249-61.

9. Nunn DN, Lory S. 1991. Product of the *Pseudomonas aeruginosa* gene *pilD* is a prepilin leader peptidase. Proc Natl Acad Sci U S A 88:3281-5.

10. Bretl DJ, Muller S, Ladd KM, Atkinson SN, Kirby JR. 2016. Type IV-pili dependent motility is co-regulated by PilSR and PilS2R2 two-component systems via distinct pathways in *Myxococcus xanthus*. Mol Microbiol 102:37-53.

11. Wu SS, Kaiser D. 1997. Regulation of expression of the *pilA* gene in *Myxococcus xanthus*. J Bacteriol 179:7748-58.

12. Perez-Burgos M, Herfurth M, Kaczmarczyk A, Harms A, Huber K, Jenal U, Glatter T, Sogaard-Andersen L. 2024. A deterministic, c-di-GMP-dependent program ensures the generation of phenotypically similar, symmetric daughter cells during cytokinesis. Nat Commun 15:6014.

13. Oklitschek M, Carreira LAM, Muratoglu M, Søgaard-Andersen L, Treuner-Lange A. 2024. Combinatorial control of type IVa pili formation by the four polarized regulators MglA, SgmX, FrzS, and SopA. J Bacteriol 206:e0010824.

14. Ward MJ, Lew H, Zusman DR. 2000. Social motility in *Myxococcus xanthus* requires FrzS, a protein with an extensive coiled-coil domain. Mol Microbiol 37:1357-71.

15. Potapova A, Carreíra LAM, Søgaard-Andersen L. 2020. The small GTPase MglA together with the TPR domain protein SgmX stimulates type IV pili formation in *M. xanthus*. Proc Natl Acad Sci U S A 117:23859-23868.

16. Mercier R, Bautista S, Delannoy M, Gibert M, Guiseppi A, Herrou J, Mauriello EMF, Mignot T. 2020. The polar Ras-like GTPase MglA activates type IV pilus via SgmX to enable twitching motility in *Myxococcus xanthus*. Proc Natl Acad Sci U S A 117:28366-28373.

17. Kaiser D. 1979. Social gliding is correlated with the presence of pili in *Myxococcus xanthus*. Proc Natl Acad Sci U S A 76:5952-6.

18. Pérez-Burgos M, Garcia-Romero I, Jung J, Schander E, Valvano MA, Søgaard-Andersen L. 2020. Characterization of the exopolysaccharide biosynthesis pathway in *Myxococcus xanthus*. J Bacteriol 202.

19. Treuner-Lange A, Chang YW, Glatter T, Herfurth M, Lindow S, Chreifi G, Jensen GJ, Søgaard-Andersen L. 2020. PilY1 and minor pilins form a complex priming the type IVa pilus in *Myxococcus xanthus*. Nat Commun 11:5054.

20. Saldias MS, Patel K, Marolda CL, Bittner M, Contreras I, Valvano MA. 2008. Distinct functional domains of the *Salmonella enterica* WbaP transferase that is involved in the initiation reaction for synthesis of the O antigen subunit. Microbiology (Reading) 154:440-453.

21. Julien B, Kaiser AD, Garza A. 2000. Spatial control of cell differentiation in *Myxococcus xanthus*. Proc Natl Acad Sci U S A 97:9098-103.

22. Guzman LM, Belin D, Carson MJ, Beckwith J. 1995. Tight regulation, modulation, and high-level expression by vectors containing the arabinose pBAD promoter. J Bacteriol 177:4121-30.

23. Marolda CL, Vicarioli J, Valvano MA. 2004. Wzx proteins involved in biosynthesis of O antigen function in association with the first sugar of the O-specific lipopolysaccharide subunit. Microbiology (Reading) 150:4095-105.

24. Herfurth M, Müller F, Søgaard-Andersen L, Glatter T. 2023. A miniTurbo-based proximity labeling protocol to identify conditional protein interactomes *in vivo* in *Myxococcus xanthus*. STAR Protoc 4:102657.

25. Datsenko KA, Wanner BL. 2000. One-step inactivation of chromosomal genes in *Escherichia coli* K-12 using PCR products. Proc Natl Acad Sci U S A 97:6640-6645.
